# An Escherichia coli FdrA Variant Derived from Syntrophic Coculture with a Methanogen Increases Succinate Production Due to Changes in Allantoin Degradation

**DOI:** 10.1128/mSphere.00654-21

**Published:** 2021-09-08

**Authors:** Nam Yeun Kim, Yeon Joo Lee, Ji Won Park, Su Nyung Kim, E Young Kim, Yuseob Kim, Ok Bin Kim

**Affiliations:** a Division of EcoScience, Graduate School, Ewha Womans University, Seoul, Republic of Korea; b Interdisciplinary Program of EcoCreative, Graduate School, Ewha Womans University, Seoul, Republic of Korea; c Department of Life Science, Ewha Womans University, Seoul, Republic of Korea; Martin Luther University of Halle-Wittenberg Institute of Biology/Microbiology

**Keywords:** succinate, glycerol fermentation, *fdrA*, allantoin degradation, oxamate

## Abstract

Wild-type Escherichia coli was adapted to syntrophic growth with Methanobacterium formicicum for glycerol fermentation over 44 weeks. Succinate production by E. coli started to increase in the early stages of syntrophic growth. Genetic analysis of the cultured E. coli population by pooled sequencing at eight time points suggests that (i) rapid evolution occurred through repeated emergence of mutators that introduced a large number of nucleotide variants and (ii) many mutators increased to high frequencies but remained polymorphic throughout the continuous cultivation. The evolved E. coli populations exhibited gains both in fitness and succinate production, but only for growth under glycerol fermentation with *M. formicicum* (the condition for this laboratory evolution) and not under other growth conditions. The mutant alleles of the 69 single nucleotide polymorphisms (SNPs) identified in the adapted E. coli populations were constructed individually in the ancestral wild-type E. coli. We analyzed the phenotypic changes caused by 84 variants, including 15 nonsense variants, and found that FdrA_D296Y_ was the most significant variant leading to increased succinate production. Transcription of *fdrA* was induced under anaerobic allantoin degradation conditions, and FdrA was shown to play a crucial role in oxamate production. The FdrA_D296Y_ variant increased glyoxylate conversion to malate by accelerating oxamate production, which promotes carbon flow through the C4 branch, leading to increased succinate production.

**IMPORTANCE** Here, we demonstrate the ability of E. coli to perform glycerol fermentation in coculture with the methanogen *M. formicicum* to produce succinate. We found that the production of succinate by E. coli significantly increased during successive cocultivation. Genomic DNA sequencing, evaluation of relative fitness, and construction of SNPs were performed, from which FdrA_D296Y_ was identified as the most significant variant to enable increased succinate production by E. coli. The function of FdrA is uncertain. In this study, experiments with gene expression assays and metabolic analysis showed for the first time that FdrA could be the “orphan enzyme” oxamate:carbamoyltransferase in anaerobic allantoin degradation. Furthermore, we demonstrate that the anaerobic allantoin degradation pathway is linked to succinate production via the glyoxylate pathway during glycerol fermentation.

## INTRODUCTION

Prokaryotes have evolved to utilize amazingly diverse energy and carbon sources in nature, as they are capable of quickly generating adaptive variants due to rapid reproduction, which accumulates mutations in a large population. This feature makes it possible to evolve prokaryotes with clear desired phenotypic changes in a controlled laboratory environment on a manageable time scale. In particular, Escherichia coli has been shown to adapt to various novel environments in experimental evolution studies, including the classical experiments by Lenski et al. (1, 2). Recent resequencing of population samples from long-term evolution experiments revealed complex genome-wide dynamics of derived alleles that underlie the continuous improvements in fitness (3).

Adaptive laboratory evolution is a powerful tool to improve microbial phenotypes via beneficial mutations (4). The applications of experimental evolution can be categorized into five general areas: (i) growth rate optimization, (ii) increase in tolerance, (iii) change in substrate utilization, (iv) increase in product yield or titer, and (v) general discovery (4). These studies predominantly used E. coli (78% among bacteria) and Saccharomyces cerevisiae (44% among yeast) (4). E. coli is an excellent model for studying adaptive evolution due to its large population size, short generation time, and feasibility of preserving strains (5, 6). For example, Jantama et al. reported that E. coli adaptively evolved over 2,000 generations after metabolic engineering to increase the production of dicarboxylic acids (7).

Succinate is a multipurpose platform chemical obtained from renewable biomass (8, 9). The global succinate market was estimated to be 132 million U.S. dollars (USD) in 2018 and is expected to reach 183 million USD by 2023, at a compound annual growth rate of 6.8% (https://www.marketsandmarkets.com/Market-Reports/succinic-acid-market-402.html). Improvement in succinate production from glycerol has been studied for over a decade, using the mainstream approach of genetic engineering to eliminate competing metabolic pathways (9–15). The bioconversion of glycerol to chemical building blocks is important to support the biofuel industry, as well as to lower production costs for succinate. Biodiesel production is expected to increase by around 4.5% annually and to reach 41 million m^3^ in 2022 (16), with the generation of 10 (wt%) glycerol as a by-product in the biodiesel production process (17).

Wild-type E. coli is limited in producing succinate from glycerol as the sole carbon source under anaerobic conditions (18, 19), as it is energetically unfavorable (1 glycerol + 1HCO_3_^−^ → 1 succinate; Δ*G*_0_′ = +385.14 kJ/mol). This means that a large proportion of glycerol should be subject to mixed-acid fermentation to allow succinate production (1 glycerol → 0.5 acetate + 0.5 ethanol + 1 formate; Δ*G*_0_′ = −134.61 kJ/mol). However, the larger is the amount of glycerol entering this energetically favorable route, the more intense is the redox imbalance of menaquinone/menaquinol (MQ/MQH_2_). When glycerol is used, the oxidation of glycerol-3-phosphate (glycerol-3-P) to dihydroxyacetone phosphate is coupled with the reduction of MQ (18), and the reoxidation of MQH_2_ requires an electron acceptor with a lower (more positive) redox potential than MQ/MQH_2_ (E_0_′ = −75 mV) such as fumarate/succinate (E_0_′ = +33 mV), dimethyl sulfoxide (DMSO)/dimethyl sulfide (DMS) (E_0_′ = +160 mV), or NO_3_^−^/NO_2_^−^ (E_0_′ = +430 mV). In the absence of an external electron acceptor, fumarate is the only usable electron acceptor and is reduced to succinate by fumarate reductase coupled with MQ/MQH_2_. Therefore, on the one hand, glycerol will be sent into the succinate branch to alleviate the redox imbalance, and the other hand, the energetics problem will be solved from outside the cell. In fact, glycerol fermentation in E. coli is able to proceed when the bacterium is cocultivated with a formate-degrading methanogen (18, 20), whereby 4 formate yields 1 methane + 3 CO_2_ + 2H_2_O (Δ*G*_0_′ = −130 kJ/mol methane). The elimination of formate, a reducing equivalent (21–24), seems to have provided the key to solving the energetics problem due to change in reaction equilibrium. In a previous paper, we showed that long-term cocultivation of E. coli with the formate degrader Methanobacterium formicicum led to increased succinate production from glycerol fermentation by E. coli (18). Here, we tracked the genetic variation in the selected E. coli strains responsible for the increased succinate production.

First, we investigated the genetic changes of E. coli that occurred during adaptation to coculture with *M. formicicum*. Genome sequencing was performed at several stages during successive cultivation to detect mutations in the genome (25, 26). A number of SNPs as the candidates responsible for adaptive changes was found. Then, we reconstructed E. coli strains carrying only one nucleotide substitution via transient mutator multiplex automated genome engineering (TM-MAGE) (27), which allowed us to identify individual mutations with significant impacts on succinate production. The most significant mutation was in *fdrA*, a gene of unknown function; therefore, it was necessary to characterize the role of FdrA to determine how it affects succinate production in E. coli. We found clues that FdrA catalyzes a global prokaryote orphan reaction for anaerobic allantoin degradation, from which we present a model pathway through glyoxylate leading to succinate production under conditions of glycerol fermentation.

## RESULTS

### Long-term E. coli and *M. formicicum* semicontinuous cocultivation.

**FIG 1:**
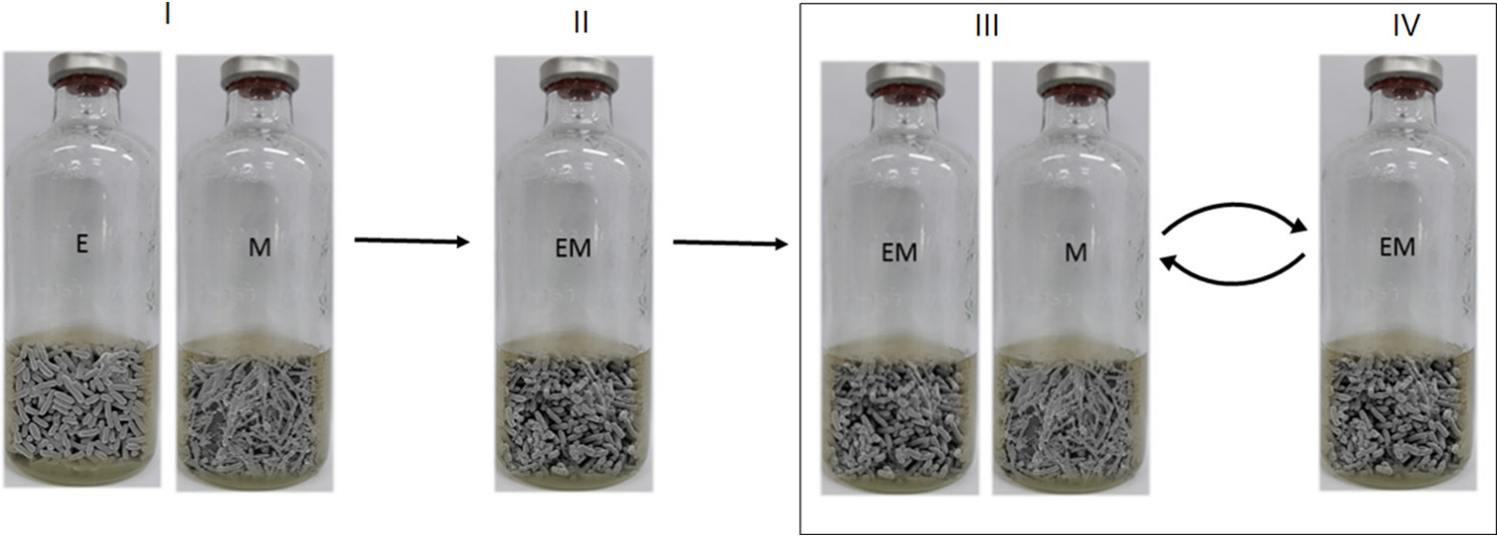
The experimental evolution scheme designed to adapt E. coli to glycerol fermentation by coculturing with *M. formicicum* under anaerobic conditions. (I) Single cultures of E. coli and *M. formicicum* grown in each of the optimal growth media; (II) coculture that inoculated culture I (10% [vol/vol] E. coli plus 30% [vol/vol] *M. formicicum*); (III) coculture that inoculated the prestage coculture II (20% [vol/vol]) plus fresh 20% (vol/vol) *M. formicicum* culture; (IV) coculture by the same inoculation as in step III. E, E. coli; M, *M. formicicum*; EM, coculture of E. coli and *M. formicicum*. The rectangle indicates that III and IV were successively repeated over 43rd cycles. The cell images were obtained by scanning electron microscopy (SEM) of the bacterial culture in this study.

In a previous paper, we reported that long-term (273-day) cocultivation of E. coli and *M. formicicum* showed increased succinate production under glycerol fermentation (18). In order to understand the genetic variation responsible for increased succinate production during cocultivation, we repeated the successive cocultivation of wild-type E. coli MG1655 with *M. formicicum* with long-term serial passages (Fig. 1) under anaerobic conditions via a gas phase of 80% H_2_ + 20% CO_2_. The presence of H_2_ imposes more stringent evolutionary pressure on E. coli as it inhibits bacterial fermentation (28). For *M. formicicum*, H_2_ + CO_2_ served as a substrate until formate was supplied by E. coli (29). One passage cycle over 1 week produced approximately three generations of E. coli cell growth. We tested four independent inoculation schemes: EM10, 10% prestage coculture; EM10M, 10% prestage coculture plus 10% fresh *M. formicicum*; EM20, 20% prestage coculture; and EM20M, 20% prestage coculture plus 20% fresh *M. formicicum*. Among them, the EM20M inoculation was optimal for continuous subcultivation, maintaining high cell density of both strains with proper ratios. The supply of additional *M. formicicum* was required because of its extremely slow growth compared to that of E. coli; without this addition, the *M. formicicum* population was diluted severely during subinoculation. Note that this fresh supply of *M. formicicum* at each transfer precluded coevolutionary changes in this partner species. The total growth of coculture EM20M, the sum of the two species’ growth, fluctuated in early passages but stabilized around the 20th round of cultivation (see Fig. S1 in the supplemental material).

10.1128/mSphere.00654-21.1 FIG S1 Growth profile of EM20M. The coculture was cultivated for 7 days on glycerol medium under anaerobic conditions. Download FIG S1, TIF file, 0.2 MB. Copyright © 2021 Kim et al. 2021 Kim et al. https://creativecommons.org/licenses/by/4.0/ This content is distributed under the terms of the Creative Commons Attribution 4.0 International license.

### Glycerol fermentation prominently increased after the 20th successive coculture.

Phenotypic changes in E. coli during the adaptation process were evaluated through analysis of product changes in the glycerol coculture fermentation. Glycerol conversion to succinate, acetate, formate, and ethanol (by E. coli) and formate conversion to methane (by *M. formicicum*) are shown in Table 1. First, E. coli glycerol consumption gradually increased and was three times higher in the 25th coculture (76 mM) than in the initial coculture (24 mM). No formate was detected, and methane was generated, indicating that the coculture functioned syntrophically, as formate was formed in amounts equal to the sum of the moles of acetate and ethanol in the E. coli fermentation but was used by *M. formicicum* and converted to methane. From the 20th passage, succinate production exceeded 20 mM, representing a >6-fold increase compared to the initial fermentation (Table 1). As a result, glycerol fermentation and succinate production increased significantly after the 20th cocultivation but not past the 30th cocultivation.

**TABLE 1:** Fermentation profiles of adapted E. coli populations during the *n*th repeated coculture^*a*^

Population	Concn (mM) of:							OD_600_	pH	Methane (ppm)	Succinate/cell mass (mM/g [dry wt])
	Glycerol added	Glycerol consumed	Succinate	Formate	Formate (calc)^*b*^	Acetate	Ethanol				
Ancestor	91.5 ± 6.9	24.0 ± 11.2	3.4 ± 1.1	0	23.9 ± 6.5	6.7 ± 0.4	17.2 ± 6.1	1.1 ± 0.1	6.9 ± 0.1	8,280.3 ± 2,707.6	9.9 ± 2.7
5th	87.6 ± 2.7	52.4 ± 6.4	10.1 ± 1.4	0	48.0 ± 4.8	10.8 ± 1.8	37.2 ± 5.4	1.2 ± 0	6.6 ± 0.1	22,921.3 ± 10,096.7	28.1 ± 4.3
15th	89.6 ± 4.9	34.9 ± 6.2	17.7 ± 1.2	0	24.1 ± 4.3	15.6 ± 3.6	8.5 ± 0.8	0.8 ± 0	6.1 ± 0.2	14,014.0 ± 2,610.2	71.1 ± 6.5
20th	85.8 ± 1.9	42.8 ± 4.1	22.0 ± 0.5	0	24.6 ± 4.3	13.7 ± 0.6	10.9 ± 3.8	0.8 ± 0.1	5.9 ± 0.1	17,558.3 ± 4,493.2	93.7 ± 7.6
25th	89.7 ± 6.3	76.7 ± 6.5	24.8 ± 1.1	0	57.9 ± 7.1	16.0 ± 0.2	41.8 ± 7.2	1.2 ± 0.1	5.5 ± 0.1	29,023.3 ± 2,982.2	69.8 ± 6.9
30th	92.3 ± 4.3	74.7 ± 8.2	26.5 ± 0.6	0	54.8 ± 9.7	13.5 ± 1.7	41.2 ± 8.0	1.2 ± 0	5.5 ± 0.1	25,675.3 ± 8,163.6	72.1 ± 1.0
39th	87.9 ± 0.8	80.8 ± 4.9	21.4 ± 1.2	0	65.8 ± 4.3	15.3 ± 0.9	50.5 ± 3.5	1.3 ± 0	5.6 ± 0.1	28,279.3 ± 2,047.3	55.0 ± 2.8
43rd	91.6 ± 6.9	76.8 ± 12.8	22.3 ± 1.1	0	61.6 ± 12.6	15.8 ± 1.0	45.7 ± 11.6	1.3 ± 0.1	5.6 ± 0.1	30,873.7 ± 14,695.5	58.8 ± 3.7

a The glycerol fermentation of E. coli was performed as coculture with *M. formicicum* for 96 h under anaerobic condition. Gene variants was analyzed using SMRT, NovaSeq 6000, and HiSeq 2500 systems. Values are averages and standard deviations (SD) for 3 replicates.

b Amount of formate generated by E. coli and immediately degraded by *M. formicicum*, which is calculated as the sum of acetate and ethanol.

### Adapted E. coli attained higher fitness in coculture with *M. formicicum* but not in single culture.

**FIG 2:**
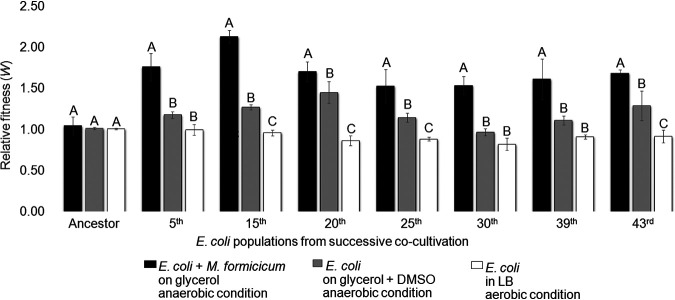
The relative fitness of evolved E. coli populations to that of the ancestor. The fitness of E. coli populations from successive coculturing was evaluated from different culture conditions: anaerobic coculture of E. coli with *M. formicicum* on glycerol (black), anaerobic single culture of E. coli on glycerol plus DMSO (gray), and aerobic single culture of E. coli in LB (white). Relative fitness was evaluated after 96 h fermentation. The different letters in E. coli populations indicate significant differences at a *P* value of <0.05. Values were determined from 3 replicates. Error bars indicate standard deviations.

Fitness of the E. coli population at several successive coculture stages was assessed by growing each successive E. coli population in competition with the initial ancestral strain. The *lacZ* gene in the ancestral strain was deleted (LMB061, Δ*lacZ*), and this was used to distinguish this strain from successive E. coli cultures via blue/white screening. It was confirmed that this *lacZ* deletion ancestral variant had no effect on fermentation (Table S1) or fitness (Table S2) under the tested conditions. The E. coli strains from successive cocultures were mixed in equal numbers with the ancestral strain and cultivated under the following three growth conditions: (i) glycerol fermentation in coculture with *M. formicicum*, (ii) glycerol fermentation in single culture with dimethyl sulfoxide (DMSO) serving as an electron acceptor (19), and (iii) aerobic growth in LB as a reference (Fig. 2; Table S2). The relative fitness value (*W*) represents the reproductive rate of a certain genotype relative to that of the ancestral genotype (1, 30, 31). A *W* value of >1 indicates that the E. coli population from that stage of successive cultivation was evolutionarily adapted to a given condition, while a *W* value of 1 indicates that no adaptation occurred.

10.1128/mSphere.00654-21.4 TABLE S1 The fermentative growth profiles of ancestral E. coli and LMB061. The E. coli strains was cocultivated with *M. formicicum* using 90 mM glycerol during 96 h. Download Table S1, DOCX file, 0.02 MB. Copyright © 2021 Kim et al. 2021 Kim et al. https://creativecommons.org/licenses/by/4.0/ This content is distributed under the terms of the Creative Commons Attribution 4.0 International license.

10.1128/mSphere.00654-21.5 TABLE S2 The relative fitness of evolved E. coli populations to that of the ancestor. The fitness of E. coli populations from the successive coculturing was evaluated after 96 h fermentation. Download Table S2, DOCX file, 0.02 MB. Copyright © 2021 Kim et al. 2021 Kim et al. https://creativecommons.org/licenses/by/4.0/ This content is distributed under the terms of the Creative Commons Attribution 4.0 International license.

In coculture with *M. formicicum*, the relative fitness of all E. coli populations that were cultivated successively (from the 5th to 43rd succession) was elevated (by 71% on average) compared to that of the ancestral E. coli LMB061 strain (Fig. 2; Table S2). The relative fitness of E. coli from the 15th successive culture was highest, with a *W* value of 2.13 (113% increase). The E. coli populations started to adapt at early stages of cocultivation. In contrast, in the single-culture glycerol fermentation with DMSO, the relative fitness of the same E. coli populations tended to be improved, but only to a minor extent (20% on average), while the 20th adaptation stage showed a significant increase, with a *W* value of 1.45 (45% increase) (Fig. 2; Table S2). The E. coli populations showed no change in fitness when they were grown aerobically in LB.

### E. coli adaptation generated hundreds of nonsynonymous variants.

To evaluate the trajectory of E. coli adaptation to methanogenic coculture, genome-wide sequence variation in the E. coli population within coculture EM20M was analyzed by pooled sequencing performed via Illumina NovaSeq 6000 and HiSeq 2500 at the 5th, 15th, 20th, 25th, 30th, 35th, 39th, and 43rd successive cocultivations. The genome of the ancestral E. coli strain was constructed by *de novo* assembly using the PacBio system and used as a reference for determining derived mutant alleles at polymorphic sites within protein-coding regions observed in the pooled sequences. Each nucleotide position of the genome was covered by a large number of aligned sequence reads (sequencing coverage of an average depth of 834), allowing base calls and allele frequencies at a high level of confidence (Table S3). Since samples in pooled sequencing were cocultures of mixed E. coli and *M. formicicum*, approximately 15 to 20% of bases that were unaligned to the reference (i.e., not found in the reference) were probably from *M. formicicum* (Table S3). We did not further analyze these sequences.

10.1128/mSphere.00654-21.6 TABLE S3 Alignment summary of whole-genome sequencing. Download Table S3, DOCX file, 0.02 MB. Copyright © 2021 Kim et al. 2021 Kim et al. https://creativecommons.org/licenses/by/4.0/ This content is distributed under the terms of the Creative Commons Attribution 4.0 International license.

The summary statistics of 1,331 variants (point mutations, insertions, and deletions) in the samples are presented in Table S4. From the allele frequency trajectories of nonsynonymous (missense) mutations (Fig. S2), we inferred that there were three episodes of simultaneous mutations at a large number of sites (between the 15th and 20th, the 25th and 30th, and the 35th and 39th cocultivations). Because allele frequencies of the mutants appearing at the same time experience change in strong correlation across multiple passages, they probably originated from a single clone in which a mutation at a certain locus dramatically increased the mutation rate at other loci genome-wide (for example, disabling the DNA repair system). It appears that each of those “mutator” alleles existed for a brief period and then was lost, because the number of variants genome-wide did not increase linearly but in discrete steps. While mutators spontaneously arising under natural conditions should be eliminated due to the load of deleterious mutations, strong selective pressure imposed by the current coculture condition is believed to have conferred fitness advantage to the observed mutators, as they contain multiple mutants that are beneficial in combination (see Discussion). In summary, the entire trajectory of variant allele frequencies shown in Fig. S2 is explained by multiple occurrences of mutator clones that increase in frequency and compete with each other. Interestingly, none of the mutator clones reached fixation in our experimental E. coli populations, suggesting a form of negative frequency-dependent selection.

10.1128/mSphere.00654-21.2 FIG S2 The allele frequency trajectories of nonsynonymous (missense) mutants. At 851 sites in total, a nonsynonymous polymorphism was observed at least once in the pooled sequencing of eight E. coli populations (5th, 15th, 20th, 25th, 30th, 35th, 39th, and 43rd passages). The estimated frequency of the derived (mutant) allele at each site is plotted. The frequencies of 69 nonsynonymous mutants that were subject to TM-MAGE analysis are highlighted in blue. Download FIG S2, TIF file, 0.4 MB. Copyright © 2021 Kim et al. 2021 Kim et al. https://creativecommons.org/licenses/by/4.0/ This content is distributed under the terms of the Creative Commons Attribution 4.0 International license.

10.1128/mSphere.00654-21.7 TABLE S4 Variant summary of whole-genome sequencing: variant counts for four mutation types (the average allele frequencies of derived alleles over those sites). Download Table S4, DOCX file, 0.02 MB. Copyright © 2021 Kim et al. 2021 Kim et al. https://creativecommons.org/licenses/by/4.0/ This content is distributed under the terms of the Creative Commons Attribution 4.0 International license.

### Seven single nucleotide polymorphisms (SNPs) led to increased succinate production in coculture but conferred no advantage in fitness.

A total of 84 nonsynonymous variants were observed in all E. coli populations in which increased succinate production was shown: E. coli of the 20th, 25th, 30th, 35th, 39th, and 43rd cocultures (Fig. S3). These 84 variants included 69 missense variants by point mutations and 15 variants causing frameshifts or stop codons. To investigate which of these 84 mutations increased succinate production, each was introduced individually into the ancestral E. coli strain. The 69 missense variants (single nucleotide substitutions) were constructed using TM-MAGE methods (27). To evaluate the 15 frameshift and nonsense variants, the corresponding 15 gene deletion mutant E. coli strains were obtained from the Keio Collection (NBRP, Japan).

10.1128/mSphere.00654-21.3 FIG S3 The nonsynonymous variants found in E. coli populations during successive cultivation. The 84 common variants were chosen for study. Download FIG S3, TIF file, 0.2 MB. Copyright © 2021 Kim et al. 2021 Kim et al. https://creativecommons.org/licenses/by/4.0/ This content is distributed under the terms of the Creative Commons Attribution 4.0 International license.

Seven of 84 variants showed very high succinate production in glycerol fermentation coculture with *M. formicicum* (Table 2; Table S5). The top seven succinate production variants had single amino acid substitutions in the genes *dnaK* (chaperone Hsp70; A586E), *ybbP* (putative ABC transporter permease; W612R), *fdrA* [putative NAD(P)-binding acyl coenzyme A (acyl-CoA) synthetase; D296Y], *pgpC* (phosphatidylglycerophosphatase C; R91L), *yfjI* (uncharacterized protein; I384V), *cysN* (sulfate adenylyl-transferase subunit 1; D171E), and *rob* (right oriC-binding transcriptional activator; R156S) (Table 2). Succinate production in each of these seven variants reached about 75% of that of the 39th coculture, which included all 84 SNPs.

**TABLE 2:** Glycerol fermentation of the single-nucleotide mutant E. coli^*a*^

Strain	Population or mutated gene	Glycerol (mM)		Fermentation product concn (mM)				OD_600_	pH	Protein (amino acid mutation)
		Added	Consumed	Succinate	Formate	Acetate	Ethanol			
Reference	Ancestor	84.7 ± 3.7	27.5 ± 5.7	3.8 ± 0.6	0	10.2 ± 3.0	19.1 ± 5.7	1.1 ± 0.1	6.8 ± 0.1	No variation
	39th	84.6 ± 4.4	69.4 ± 9.9	20.1 ± 1.6	0	16.2 ± 2.6	39.7 ± 11.2	1.2 ± 0.1	5.8 ± 0.1	Contained in all 84 variations
Single-nucleotide mutant E. coli	*dnaK* (b0014)	79.7 ± 1.7	73.2 ± 3.5	13.9 ± 0.9	0	20.3 ± 5.4	43.8 ± 3.8	1.1 ± 0	6.1 ± 0.1	Chaperone Hsp70 (A586E)
	*ybbP* (b0496)	85.4 ± 3.3	77.3 ± 3.1	14.5 ± 1.3	0	22.2 ± 6.7	48.9 ± 8.1	1.2 ± 0.1	6.0 ± 0.2	Putative ABC transporter permease (W612R)
	*fdrA* (b0518)	85.1 ± 6.0	64.9 ± 3.2	16.4 ± 1.0	0	21.0 ± 4.1	33.5 ± 7.7	1.0 ± 0	6.0 ± 0.1	Putative NAD(P)-binding acyl-CoA synthetase (D296Y)
	*pgpC* (b2560)	90.5 ± 3.5	45.0 ± 2.6	16.0 ± 0.6	0	19.7 ± 0.6	15.1 ± 1.6	0.8 ± 0	6.1 ± 0.1	Phosphatidylglycerol phosphatase C (R91L)
	*yfjI* (b2625)	86.1 ± 5.0	73.6 ± 1.4	14.8 ± 0.4	0	24.1 ± 1.7	41.5 ± 1.1	1.1 ± 0.1	5.9 ± 0.1	Uncharacterized protein (I384V)
	*cysN* (b2751)	82.8 ± 5.4	77.3 ± 3.0	14.7 ± 0.2	0	20.3 ± 6.4	50.2 ± 4.2	1.2 ± 0.1	6.1 ± 0.1	Sulfate adenylyltransferase subunit 1 (D171E)
	*rob* (b4396)	83.9 ± 5.0	74.7 ± 7.0	14.1 ± 2.2	0	16.1 ± 7.2	51.3 ± 11.7	1.1 ± 0.1	6.1 ± 0.1	Right oriC-binding transcriptional activator (R156S)

a The glycerol fermentation of E. coli was performed as coculture with *M. formicicum* for 96 h under anaerobic condition. Seven E. coli strains were selected after evaluating the fermentation of a total 84 mutant strains, and they accounted for more than 75% of the succinate production observed in the 39th population. Values are averages and SD for 3 replicates.

10.1128/mSphere.00654-21.8 TABLE S5 Glycerol fermentation of 77 mutant E. coli strains as cocultures with *M. formicicum*. The fermentation profiles were evaluated after 96 h fermentation. Download Table S5, DOCX file, 0.03 MB. Copyright © 2021 Kim et al. 2021 Kim et al. https://creativecommons.org/licenses/by/4.0/ This content is distributed under the terms of the Creative Commons Attribution 4.0 International license.

**FIG 3:**
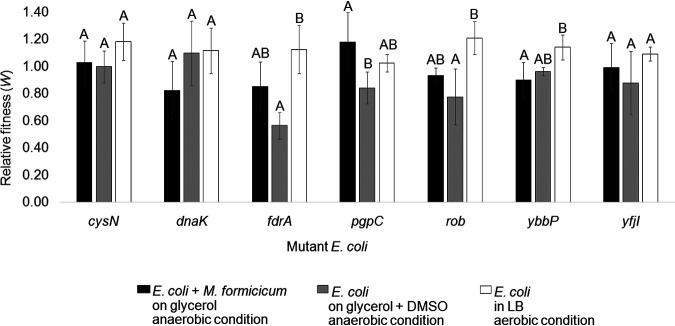
The relative fitness of single-nucleotide mutant E. coli strains to that of the ancestor. The fitness of E. coli mutants was evaluated from different culture conditions: anaerobic coculture with *M. formicicum* on glycerol (black), anaerobic single culture on glycerol plus DMSO (gray), and aerobic single culture in LB (white). Relative fitness was evaluated after 96 h fermentation. The different letters in E. coli populations indicate significant differences at a *P* value of <0.05. Values were obtained with three replicates. Error bars indicate standard deviations.

When the relative fitness of the seven variants was measured under the growth conditions shown in Fig. 2 for evolved populations, no significant increase was found (Fig. 3; Table S6). The majority of variants with lower fitness than the ancestor were observed under the anaerobic coculture condition. In addition, the relative fitness of the variants was measured under other growth conditions (aerobic growth on glycerol, xylose, glucose, or succinate; anaerobic growth on xylose, glucose, glycerol plus DMSO, or glycerol plus fumarate), but we could not find significant changes (Table S7).

10.1128/mSphere.00654-21.9 TABLE S6 Fitness of single-nucleotide mutant E. coli strains relative to that of the ancestor. The fitness of E. coli mutants was evaluated after 96 h fermentation. Download Table S6, DOCX file, 0.02 MB. Copyright © 2021 Kim et al. 2021 Kim et al. https://creativecommons.org/licenses/by/4.0/ This content is distributed under the terms of the Creative Commons Attribution 4.0 International license.

10.1128/mSphere.00654-21.10 TABLE S7 Relative fitness and cell growth of mutant populations depending on culture conditions using several substrates in M9 medium. Ancestor and 39th-passage E. coli populations were used as comparative controls. (A) Relative fitness on aerobic culture; (B) relative fitness on anaerobic culture; (C [a to d]) cell density determined at 600 nm on aerobic glycerol, xylose, glucose, and succinate cultures; (D [a to d]) cell density determined at 600 nm on anaerobic cultures with glycerol plus DMSO, xylose, glucose, and glycerol plus fumarate. The relative fitness was evaluated after 24 h culture. Each substrate was added at 50 mM. Values were determined for 3 replicates. Download Table S7, DOCX file, 0.02 MB. Copyright © 2021 Kim et al. 2021 Kim et al. https://creativecommons.org/licenses/by/4.0/ This content is distributed under the terms of the Creative Commons Attribution 4.0 International license.

In summary, 7 missense mutations were found to lead to increases in succinate production, but none conferred an advantage in fitness.

### The *fdrA* variant increases succinate production by altering the allantoin pathway.

**FIG 4:**
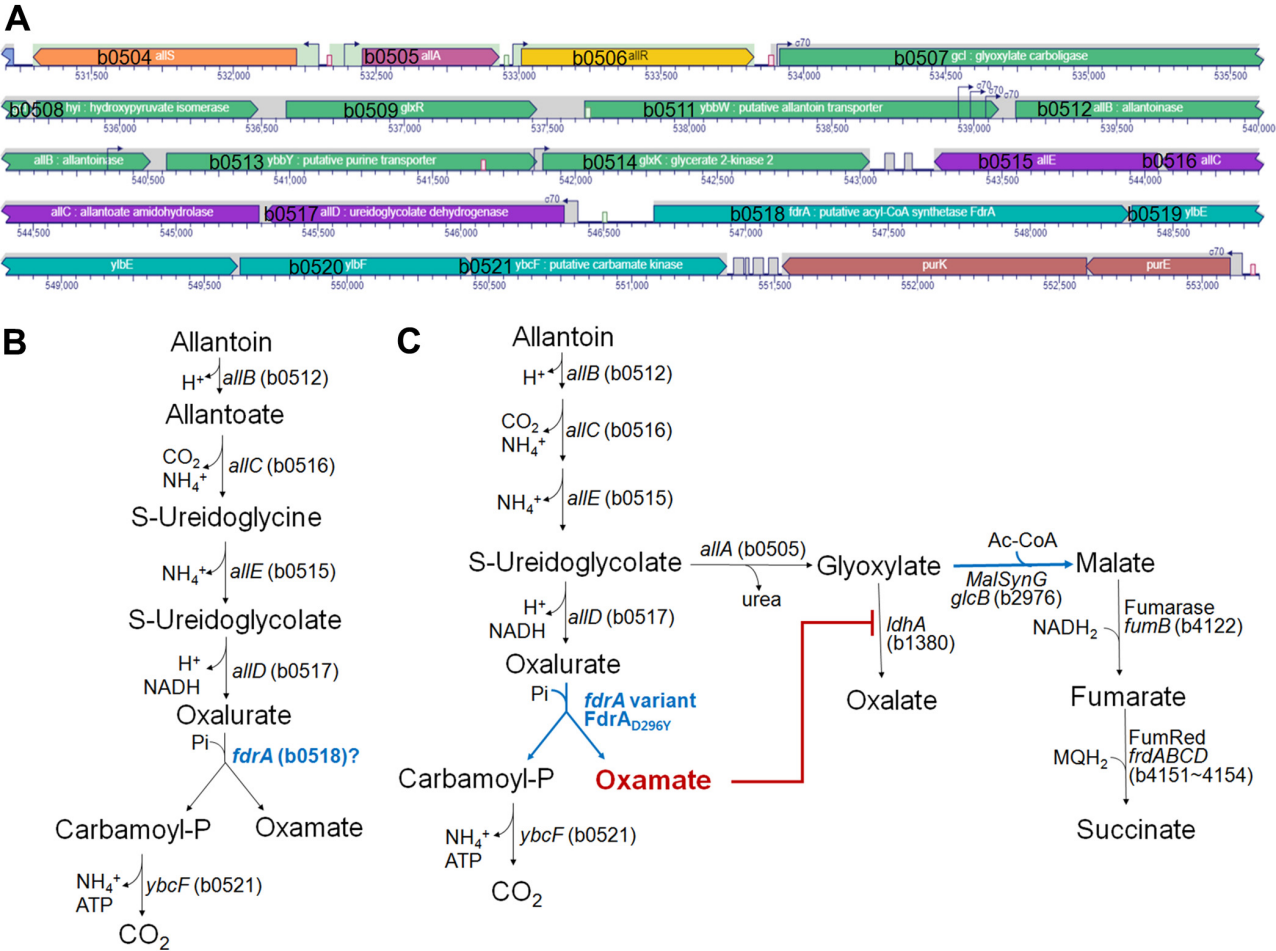
Anaerobic allantoin pathway. (A) Gene clusters in the chromosome for anaerobic allantoin degradation. Map positions 513,200 to 553,200 in the chromosome of MG1655 were extracted from Genome Browser of the EcoCyc E. coli database and modified. (B) Allantoin degradation pathway in which *fdrA* encodes the enzyme responsible for the step splitting oxalurate into oxamate and carbamoyl phosphate. (C) Succinate production via the glyoxylate pathway during glycerol fermentation.

The *fdrA* variant showed the highest succinate production (Table 2); therefore, we examined the function of this gene and how its genetic variation affects fermentation. In E. coli, *fdrA* (b0518) is located immediately next to a cluster of genes responsible for anaerobic allantoin degradation (b0504 to b0517) (Fig. 4A). *fdrA* encodes a 555-amino-acid polypeptide containing domains similar to those of the SucD superfamily (COG0074) and ligase CoA superfamily (Pfam 00549) based on an NCBI Conserved Domain Database (CDD) search (https://www.ncbi.nlm.nih.gov/Structure/cdd/wrpsb.cgi). In that context, oxamate:CoA ligase, oxalate:CoA ligase, and succinate:CoA ligase were considered possible functions for the gene.

The promoter region of *fdrA* was fused with the *lacZ* reporter gene, and the possible effector (inducer) was explored by β-galactosidase assays (Table 3). Under anaerobic conditions, allantoin greatly increased the transcription of *fdrA*, while oxamate and oxalate had no effect on gene expression (Table 3). Under aerobic conditions, the presence of succinate, oxalate, oxamate, or glycerol did not induce the transcription of *fdrA* (data not shown). Thus, while FdrA plays a role in anaerobic allantoin degradation, oxamate, succinate, and oxalate are not expected to be substrates.

**TABLE 3:** Transcriptional regulation of *fdrA-lacZ* reporter gene fusion in pMW151 contained in E. coli MC4100

Effector (20 mM)	β-Galactosidase activity (MU)^*a*^
Allantoin	9,511.1 ± 1,062.6
Oxamate	71.3 ± 19.9
NH_4_Cl	72.8 ± 37.3
Oxalate^*b*^	85.4 ± 54.1

a The bacteria were grown on glycerol (50 mM) and DMSO (50 mM) in M9 medium to an OD_600_ of 0.4 to 0.6 under anaerobic conditions. Cultures contained 20 mM allantoin, oxamate, NH_4_Cl, or oxalate as the effector. The β-galactosidase activity of the empty *lacZ* fusion plasmid pJL29 was measured for each culture condition and as a blank value. Values are averages and SD, derived from at least three independent cultures, each measured in quadruplicate. MU, Miller units.

b eM9 (enriched M9 medium) was used for cultivation.

Changes in anaerobic allantoin degradation in the *fdrA* mutant were analyzed (Table 4). In the wild-type strain, most of the allantoin was degraded into oxamate (∼80%) and a small fraction into oxalate (∼20%) (Table 4). In the Δ*fdrA* strain, no oxamate was produced; instead, the oxalate fraction increased. The *fdrA* cloned plasmid (pNTR-SD::*fdrA*) was able to complement oxamate production with the decrease in oxalate production. These data show that FdrA catalyzes the step producing oxamate and indicate that oxalate is linked to an alternative path of oxamate production in a metabolic context.

**TABLE 4:** Effect of *fdrA* variation on anaerobic allantoin degradation

Strain	Allantoin consumed (mM)	Production (mM)		Glycerol consumed (mM)	Production (mM)				Growth	pH
		Oxamate	Oxalate		Succinate	Formate	Acetate	Ethanol		
MG1655 (ancestor)	21.1 ± 1.0^*a*^	16.6 ± 1.2	3.2 ± 1.4	29.4 ± 1.2	6.2 ± 0.7	26.8 ± 0.4	15.3 ± 1.0	12.6 ± 0.6	0.7 ± 0	6.2 ± 0.1
MG1655 Δ*fdrA*	21.0 ± 0.9	0	10.9 ± 0.8	28.6 ± 1.2	5.4 ± 0.8	27.2 ± 1.8	14.4 ± 0.6	13.7 ± 0.6	0.6 ± 0	6.1 ± 0.1
MG1655 Δ*fdrA* pNTR-SD::*fdrA*	20.2 ± 0.2	5.6 ± 1.3	7.9 ± 0.2	28.5 ± 0.7	6.3 ± 0.1	20.3 ± 0.6	7.5 ± 0.2	12.0 ± 0.1	0.6 ± 0	6.0 ± 0
MG1655 *fdrA*_D296Y_	20.8 ± 0.8	20.9 ± 1.7	0.4 ± 0.8	36.3 ± 2.0	10.0 ± 0.3	30.0 ± 1.6	20.3 ± 0.4	11.9 ± 0.6	0.6 ± 0	5.9 ± 0

a Bacterial strains (wild type, FdrA_D296Y_, Δ*fdrA*, and Δ*fdrA* containing pNTR-SD::*fdrA*) were anaerobically grown in M9 containing glycerol (50 mM), DMSO (50 mM), and allantoin (20 mM) for 72 h, whereby allantoin was used as a sole nitrogen source. Values are averages and SD for 3 replicates.

In the *fdrA* variant (encoding the D296Y amino acid substitution), almost all allantoin was degraded into oxamate and a negligible amount into oxalate (Table 4), suggesting that the D296Y amino acid substitution enhances enzyme activity to produce oxamate or increased substrate affinity (for oxalurate). The 25% increase in oxamate production could be considered the direct cause of improvement in succinate production in this FdrA_D296Y_ variant.

In summary, *fdrA* encodes an enzyme playing a role in the degradation of allantoin (oxalurate) into oxamate. The FdrA_D296Y_ mutant accelerates the conversion of allantoin into oxamate, which improves succinate production.

## DISCUSSION

### Evolutionary changes driven by mutators.

While E. coli was cocultivated continuously with *M. formicicum*, a clear increase in succinate yield in the 39th round of EM20M coculture was confirmed. We therefore attempted to identify genetic variants of the E. coli population at this time point by whole-genome pooled sequencing. Surprisingly, a large number of nucleotide variants in the protein-coding sequences was found at high frequencies. We subsequently analyzed the allele frequency trajectories of these variants by sequencing the coculture samples before (5th, 15th, 20th, 25th, and 35th) and after (43rd) the 39th passage. As shown in Fig. S2, derived alleles entered the population in large clusters, and the frequency trajectories of alleles in each cluster fluctuated in a highly correlated manner. This pattern suggests that the variants were generated in clones that experienced dramatic increases in mutation rate (“mutators”). Mutators arise spontaneously under natural conditions and were observed frequently in other E. coli experimental evolution studies (3, 32, 33). Because a large number of mutations arising in mutators are deleterious, mutators will be eliminated from the population under normal growth conditions. However, under a harsh condition for survival, as imposed in our coculture experiment and other studies, a modifier of the genomic mutation rate (e.g., a defect in the DNA repair system) can increase to a high frequency by hitchhiking along with multiple variants at other loci, some of which provide a fitness advantage under the condition. In our experiment, each mutator clone appears to have lost its mutation rate modifier, because the total number of variants in the E. coli population did not increase steadily but in discrete steps (Fig. S2; Table S4). It is possible that a heavy load of deleterious mutations that quickly accumulate upon the emergence of the mutator clone selected for the ancestral allele of the modifier locus by reverse mutation.

Another interesting pattern of the allele frequency trajectories is that the variants remained polymorphic (without fixation in any locus) throughout the experiment, even though many of them reached frequencies close to 1. This suggests the presence of negative frequency-dependent selection acting on the ancestral and novel (mutator-established) clades. Such negative frequency-dependent selection was prevalent in experimental populations of E. coli (3). At this point, however, we are not aware of any mechanism that causes frequency dependence within our E. coli population. The unique evolutionary dynamics observed in our experiment—multiple entrance of brief mutators and balanced polymorphism among clones—might be the result of the unusually large population size of E. coli. The size of our E. coli population decreased only 5-fold during transfer to deal with poor growth under the coculture condition with the methanogen, while other experimental evolution studies use at least 100-fold dilutions at serial transfer. Because a population bottleneck at dilution determines the effective population size, we effectively maintained a continuous culture that contained a large E. coli population. A large population size promotes the generation of diverse mutants, including mutators that accrue particular mutations at other loci that are beneficial in combination, and increases the strength of natural selection that might be needed for persistence of the ancestor clone (34).

### FdrA is an oxamate-producing enzyme in anaerobic allantoin degradation.

The NCBI CDD search presented a putative succinyl-CoA synthetase (SCS) as the strongest enzyme candidate encoded by the *fdrA* gene. We detected no SCS activity from FdrA expressed by pCA245N::*fdrA* (overexpression) or pNTR-SD::*fdrA* (complementation) using an SCS activity colorimetric assay kit (Sigma) (data not shown). In addition, the *fdrA*-*lacZ* fusion showed no induction with succinate or its related substrate (data not shown), indicating that *fdrA* does not encode an SCS or a succinate-related enzyme.

Smith et al. selected *fdrA* (b0518) and *ylbF* (b0520) as candidate genes for oxamate:carbamoyltransferase (OXTCase) using a bioinformatics strategy, even though neither predicted protein had sequence similarity to known carbamoyltransferases (Fig. 4A) (35). OXTCase is an orphan enzyme whose activity is required in anaerobic allantoin degradation, resulting in carbamoyl phosphate and oxamate (Fig. 4B) (35). The predicted *ylbF* gene product was proposed as the best candidate for OXTCase, and *fdrA* was assumed to encode an oxamate:CoA ligase, which subsequently removes oxamate, since *fdrA* encodes a predicted conserved domain for CoA-ligase activity (35). However, the anaerobic cultivation in this study showed that E. coli does not consume oxamate (data not shown). In addition, the *fdrA*-*lacZ* fusion was induced highly by allantoin, with no inductive effect in the presence of oxamate (Table 3). Thus, it appears that *fdrA* does not encode an oxamate:CoA ligase. Furthermore, the Δ*fdrA* strain could not produce oxamate in allantoin degradation analysis (Table 4), indicating that *fdrA* encodes an enzyme producing and not degrading oxamate. Taken together, these findings indicate that *fdrA* is expected to encode the enzyme responsible for the step splitting oxalurate into oxamate and carbamoyl phosphate. Therefore, FdrA is likely the orphan OXTCase enzyme or a new unique enzyme, and further research is needed to reach a conclusion.

### Increased succinate production by FdrA_D296Y_.

According to the analysis in Table 4, anaerobic allantoin degradation during glycerol fermentation appears to branch after ureidoglycolate (Fig. 4C), where ureidoglycolate is converted to oxalate via glyoxylate. Ureidoglycolate lyase (*allA*, b0505) removes urea from ureidoglycolate and converts it to glyoxylate (36), which most likely is oxidized to oxalate by lactate dehydrogenase (LDH) (37). LDH exhibits a broad substrate spectrum including pyruvate, 2-keto acids, and glyoxylate (38). In the Δ*fdrA* strain, the oxamate route is blocked due to the absence of FdrA (probably OXTCase), and more ureidoglycolate flows into the glyoxylate pathway, leading to oxalate (Table 4). The variant FdrA_D296Y_ accelerates oxamate production (Table 4; Fig. 4C), which is an effective inhibitor of LDH (39). This fast-generated oxamate completely inhibits LDH to prevent oxalate production from glyoxylate (Table 4). As a result, more glyoxylate is exposed to malate synthase G, increasing the succinate level (Fig. 4C). There are two malate synthases, A (acetate type; *aceB* [b4014]) and G (glycolate type; *glcB* [b2976]), the latter of which is induced during growth on glycerol as the sole carbon source (40, 41).

Nitrogen content of the medium used for the present syntrophic cultivation was very low. The N limitation was sufficient to induce purine degradation, resulting in allantoin production, which would have been degraded immediately to nitrogens and oxamate (42).

Altogether, FdrA functions as an OXTCase or the oxamate-producing enzyme in E. coli, and its variant FdrA_D296Y_ accelerates oxamate production. The increase in oxamate results in increased succinate production by flowing more glyoxylate to the C4 branch via malate synthase G by inhibiting LDH.

## MATERIALS AND METHODS

### Adaptation and fermentation.

E. coli K-12 MG1655 (F^–^ λ^–^ *ilvG rfb-50 rph-1*) was used as the ancestral strain for adaptation and was grown anaerobically at 37°C in LB broth (Difco, USA) up to an optical density at 600 nm (OD_600_) of 1.20. *M. formicicum* JF-1 was obtained from the German Type Culture Collection (DSMZ, Germany) and anaerobically cultivated at 37°C in DSMZ 119 medium to an OD_600_ of 0.27.

To adapt E. coli to glycerol fermentation with *M. formicicum*, the cocultivation of E. coli and *M. formicicum* was subcultured continuously, where each cocultivation took 7 days. The coculture was performed in 100 ml adaptation medium with 70 mM glycerol and cultivated anaerobically under an 80% H_2_–20% CO_2_ gas mixture at 37°C. The adaptation medium contained 3 mM KH_2_PO_4_, 1 mM K_2_HPO_4_, 4 mM NH_4_Cl, 5 mM KCl, 6 mM NaCl, 1 mM MgCl_2_, 21 mM HCO_3_Na, 5 mM CO_3_Na_2_, 0.2 mM C_6_H_7_NaO_6_, 5.1 mM CaCl_2_, 10 ml NB trace mineral solution (43), 1.0 ml selenite-tungstate solution (13 mM NaOH, 17 μM Na_2_SeO_3_, and 12 μM Na_2_WO_4_), 10 ml vitamin solution (DSMZ, media 141), 0.1% (wt/vol) yeast extract, 1 mM cysteine, and 2 μM resazurin. The pH value was adjusted to 7.0. To constitute a parent coculture of E. coli and *M. formicicum*, 10% (vol/vol) E. coli and 30% (vol/vol) *M. formicicum* were cultured in the adapted medium for 7 days and used in a successive repeat culture. Twenty percent of the prestage coculture was inoculated into fresh medium. An additional fresh 20% (vol/vol) *M. formicicum* then was added.

Glycerol fermentation was performed at 37°C for 96 h under anaerobic conditions with an 80% N_2_–20% CO_2_ gas mixture in 100 ml of fermentation medium. Ten percent (vol/vol) E. coli and 30% (vol/vol) *M. formicicum* were inoculated into the medium with 90 mM crude glycerol (Aekyung Petrochemical, Republic of Korea). The fermentation medium contained 1.5 mM KH_2_PO_4_, 2.3 mM K_2_HPO_4_, 9.4 mM NH_4_Cl, 2 mM MgSO_4_, 2 mM CaCl_2_, 38.8 mM NaCl, 0.01 mM FeSO_4_, 20 mM HCO_3_Na, SL-10 (DSMZ, medium 320), 10 ml vitamin solution (DSMZ, medium 141), 0.1% (wt/vol) yeast extract, 0.2% (wt/vol) Casitone, 1.7 mM cysteine, 1.3 mM Na_2_S, and 2 μM resazurin.

### Ancestor library construction.

Construction of the ancestral E. coli library was performed at DNA Link, Inc. (Republic of Korea). The constructed library was validated with an Agilent 2100 Bioanalyzer. Ancestor E. coli was prepared by P6-C4 chemistry and sequenced using one SMRT cell (Pacific Biosciences of CA, USA) with a MagBead OneCellPerWell v1 protocol (insert sizes, 20 kb; movie time, 1 × 240 min) (44). *De novo* assembly of ancestor E. coli was conducted using the hierarchical genome assembly process (HGAP, version 2.3) (45). As the estimated genome size was 4,637,247 bp, and average coverage was 127-fold; error correction was performed based on the longest of about 30-fold (441,311,609 bp) seed bases, with the rest showing shorter reads. As a result of the HGAP, 4,637,247-bp *N*_50_ contig and 4,637,247-bp total contig lengths were obtained. Finally, forms for each contig were assessed using MUMmer 3.5 (45), and one of the self-similar ends was trimmed for manual genome closure. Putative gene coding sequences (CDSs) from the assembled contigs were identified using Glimmer v3.02 (46). Open reading frames (ORFs) were searched using BLAST alignment (https://www.ncbi.nlm.nih.gov/books/NBK1762/). Gene Ontology (GO) annotation was assigned to each of the ORFs by Blast2GO software (47, 48) analyzing the best hits of the BLAST results. Additionally, rRNAs and tRNAs were predicted using RNAmmer 1.2 and tRNAscan-SE 1.4 (49), respectively.

### Whole-genome sequencing and variant call analysis.

Cocultures preserved in 20% glycerol at −80°C were used to analyze the whole E. coli genome. The analysis was performed at DNA Link (Republic of Korea). High-molecular-weight genomic DNA was sheared randomly to yield DNA fragments using the Covaris S2 system. The fragments were blunt ended and phosphorylated, and a single A nucleotide was added to the 3′ ends of the fragments in preparation for ligation to an adapter with a single-base T overhang. Adapter ligation at both ends of the genomic DNA fragment conferred different sequences at the 5′ and 3′ ends. Ligated DNA was PCR amplified to enrich for fragments. After quantitative PCR (qPCR) using SYBR green PCR master mix (Applied Biosystems, USA), libraries that were index tagged in equimolar amounts in the pool were combined. The whole genome was analyzed using a NovaSeq 6000 and HiSeq 2500 system (Illumina, USA) for 2 × 100 sequencing. Sequence quality control (QC) was performed through FastQC 0.11.5 (https://www.bioinformatics.babraham.ac.uk/projects/fastqc/), and the sequence was mapped to the ancestor E. coli reference genome sequence from the results of *de novo* assembly using bwa 0.7.12 (50). BAM files were sorted, and duplicates were marked using PICARD 2.2.1. Variants were called and filtered using UnifiedGenotyper of the Genome Analysis Toolkit (51). Then, the variants were annotated with effects and genotypes with SnpEff (52).

### High-performance liquid chromatography analysis.

Culture supernatants were analyzed using the high-performance liquid chromatography (HPLC) LaChrom Elite system (Hitachi High Technologies, Japan), comprising an L-2130 pump, an L-2350 column oven, an L-2200 autosampler, and an Aminex HPX-87H ion exclusion column (300 mm by 7.8 mm; Bio-Rad, USA). The mobile phase was 4 mM H_2_SO_4_ supplied at a constant flow rate of 0.55 ml/min. The quantitative determination was carried out using an L-2490 refractive index detector and L-2400 UV detector (210 nm).

### Quantification of methane via gas chromatography.

Methane from the air column of the culture was analyzed using a 6500 GC system (YL Instruments, Republic of Korea). Gas extracted from the air column was injected and separated using a Carboxen 1006 PLOT column (30 m by 0.53-mm inside diameter [i.d.]; Sigma-Aldrich, USA). Methane was quantified using a flame ionization detector (YL Instruments, Korea).

### Construction of E. coli mutants with base substitutions.

Chromosomal base substitutions in E. coli were constructed using the transient mutator multiplex automated genome engineering (TM-MAGE) method (27) with some modifications. Oligonucleotides for TM-MAGE were designed using MODEST (53) and were set to 90 bp in length containing 15-bp minimum end homology and 50-bp long-insertion end homology. All oligonucleotides were synthesized by Bioneer (Korea) with two phosphorothioated bases introduced at the 5′ termini and purified by PAGE. Ancestor E. coli analyzed by next-generation sequencing (NGS) was used as a starter strain for TM-MAGE. The plasmid pMA7-SacB (Addgene, USA) was used to transform ancestor E. coli. The E. coli harboring pMA7-SacB was grown overnight in LB (Lennox, Sigma-Aldrich, USA) containing 100 μg/ml ampicillin and 0.5 mM MgSO_4_ at 37°C with 180 rpm shaking. The main culture was inoculated with 1% overnight culture in the same medium and cultivated under the same condition. When the OD_600_ reached 0.4 to 0.6, l-arabinose was added to a final concentration of 0.2% (wt/vol), cultivation was continued for 10 min, and the culture was incubated on ice for 15 min. Bacterial cells were harvested by Combi 514-R centrifugation (Hanil, Republic of Korea) at 4,000 × *g* for 5 min at 4°C, washed three times using cooled distilled water (30 ml, 1 ml, and 1 ml), and concentrated by final resuspension with 200 μl cooled distilled water. The mixture of competent cells (50 μl) and MAGE oligonucleotide (0.5 μl; 50 pmol) was electroporated using a MicroPulser (Bio-Rad, USA), resuspended in LB (Lennox, Sigma-Aldrich, USA) containing 100 μg/ml ampicillin and 0.5 mM MgSO_4_, and spread on an agar plate. Individual colonies were picked and selected by multiplex allele-specific colony (MASC) PCR (54). Positive colonies were streaked on sucrose plates (10 g/liter tryptone, 5 g/liter yeast extract, 15 g/liter agar, and 5% [wt/vol] sucrose) for plasmid curing. Then, successful point mutations in the genome were confirmed by DNA sequencing (Cosmogenetech, Republic of Korea).

### Relative fitness assays.

Relative fitness of E. coli strains was evaluated by measuring competitive growth when the strains and ancestor E. coli were cocultivated. Fitness was tested under three culture conditions: (i) anaerobic coculture of E. coli and *M. formicicum* on glycerol (90 mM), (ii) anaerobic single-culture of E. coli on glycerol (90 mM) plus DMSO (50 mM), and (iii) aerobic single-culture of E. coli in LB. Five percent (vol/vol) of each competing E. coli strain and 30% (vol/vol) *M. formicicum* were inoculated into the medium. Anaerobic cultures were performed for the fermentation medium (100 ml) for 96 h. Aerobic culture in LB (10 ml) was used as the reference condition, and aerobic cultures were incubated for 24 h after inoculation at 0.5% (vol/vol) with each of the competing E. coli strains.

After cultivation, bacterial cells were diluted to form colonies ranging in number from 50 to 100 on X-Gal (5-bromo-4-chloro-3-indolyl-β-d-galactopyranoside) agar plates. The two competing strains were distinguished by white/blue colonies. The ancestral E. coli (Δ*lacZ*) strain was white, and the competing strain was blue. Relative fitness (*W*) was calculated using the ratio of Malthusian parameters of two strains: *W* = ln(*A*_f_/*A*_i_)/ln(*B*_f_/*B*_i_), where *A* indicates the cell density of the evolved or mutated population, *B* indicates the cell density of the ancestral population, and subscripts i and f indicate initial and final densities, respectively (1, 30, 31). A *W* value of >1.0 indicates that the fitness of the E. coli population increased compared to that of the ancestor, while a *W* value of <1.0 indicates that fitness decreased.

### Construction of the *fdrA-lacZ* reporter gene fusion.

Fusion of the *fdrA* promoter region with *lacZ* was constructed using the reporter gene fusion plasmid pJL29 (J. Lucht and E. Bremer, unpublished data). The *fdrA* promoter region was amplified using primers fdrA_SalI (5′-CGG CAT TGT CGA CAT GTA AA-3′) and fdrA_HindIII (5′-CCT CTT CAA GCT TCT GCA TA-3′) from E. coli MG1655 genomic DNA. The corresponding fragments covered the complete upstream regulatory regions of *fdrA* and were cloned into the corresponding restriction sites of the reporter gene fusion plasmid pJL29. The *fdrA-lacZ* gene fusion was used to transform E. coli MC4100, which is a *lacZ*-deficient strain.

### β-Galactosidase activity assay.

Bacteria were grown anaerobically in M9 or eM9 medium, which is M9 minimal medium supplemented with acid‐hydrolyzed casein and l‐tryptophan (55). Glycerol (50 mM) and DMSO (50 mM) were used as the carbon sources. Cultures contained 20 mM allantoin, oxamate, NH_4_Cl, or oxalate as the effector. The subcultures (5%) were inoculated into the main culture. The growth range for the assay was from an OD_600_ of 0.4 to 0.6. The β-galactosidase activity was determined as described by Miller (56). Empty pJL29 vector was used to remove background values of β-galactosidase activity. The activities were determined using three independent growth experiments.

### Inactivation and complementation of *fdrA*.

The *fdrA* gene was deleted using the method of Datsenko and Wanner (57). For insertional inactivation, the PCR product of the *cat* chloramphenicol resistance gene from pKD3 was used and was flanked by FRT sequences. Primers fdrA_H1P1 (5′-ATT AAC ACT GCT CGT GCA ATT GCC ATG GGT GCA ATT TTT AAG GAG TTG TTT GTG TAG GCT GGA GCT T-3′) and fdrA_H2P2 (5′-TCG ATA ACC GCA TTG GCT TGC GCC ACT GAT GTA AAC ATG GGA ACC CC C CAT ATG AAT ATC CTC CTT A-3′) contain parts of the regions adjacent to FRT and the 5′ and 3′ regions of amplified *fdrA*, respectively. The PCR products were purified, concentrated, and used for transformation. Chloramphenicol-resistant colonies were tested to determine loss of the helper plasmid (pKD46) by examining ampicillin sensitivity. To delete *cat*, the mutant was transformed with the FLP helper plasmid pCP20 and selected at 30°C (58). The plasmid pNTR-SD::*fdrA* (156#6; NBRP, NIG, Japan) was used with 0.2 mM IPTG (isopropyl-β-d-thiogalactopyranoside) to complement the *fdrA* inactivated strain.

### Statistical analysis.

Statistical analyses were performed using PASW Statistics 18 (SPSS, Inc.). Data were analyzed using one-way analysis of variance (ANOVA). *Post hoc* analysis was performed using Duncan’s multiple-comparison test. Statistical significance was defined as a *P* value of <0.05.
