# Supplementary figures and images for: An Escherichia coli FdrA Variant Derived from Syntrophic Coculture with a Methanogen Increases Succinate Production Due to Changes in Allantoin Degradation
Source: mSphere. 2021 Sep 8;6(5):e00654-21. doi: 10.1128/mSphere.00654-21 (PMC8550087; doi:10.1128/mSphere.00654-21)

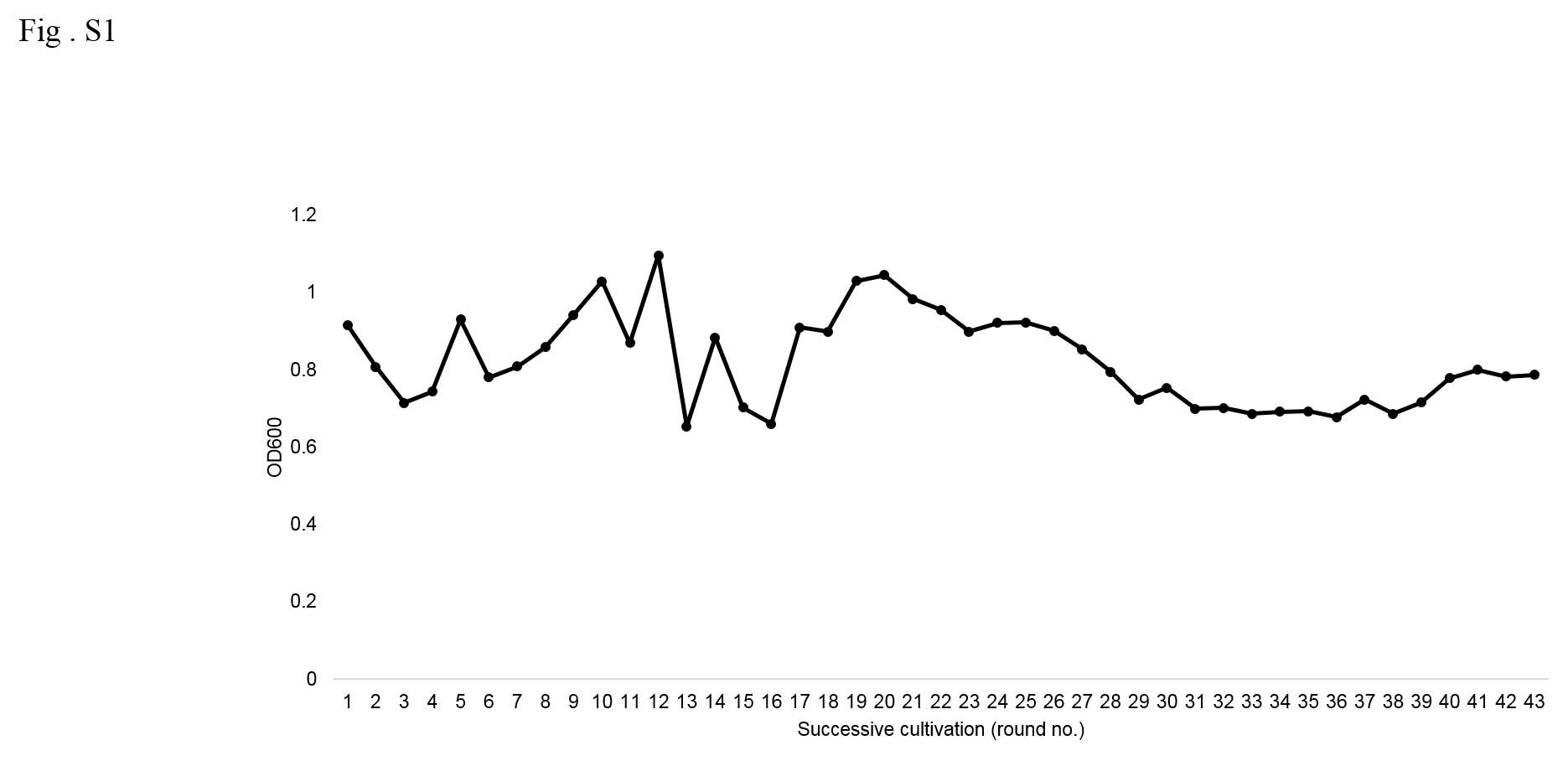

Supplement: FIG S1 [file msphere.00654-21-sf001.tif]

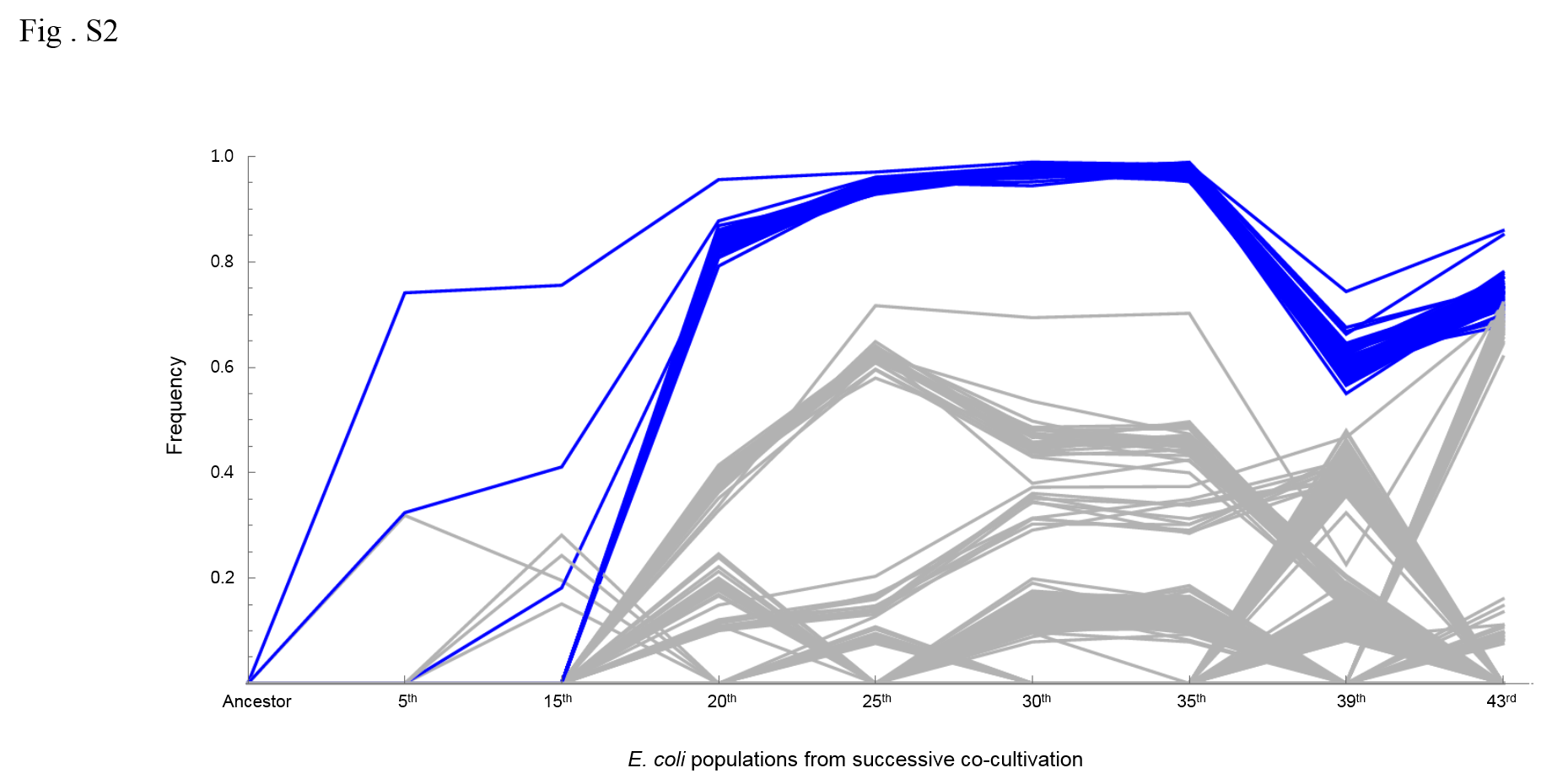

Supplement: FIG S2 [file msphere.00654-21-sf002.tif]

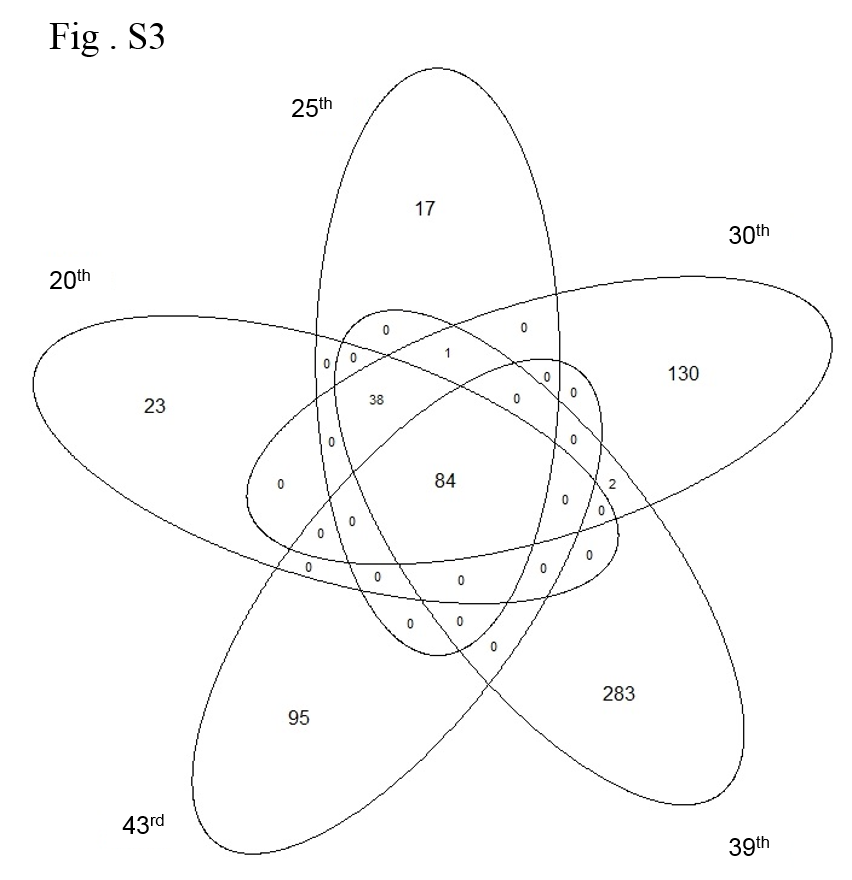

Supplement: FIG S3 [file msphere.00654-21-sf003.tif]
