# Supplementary material for: An Escherichia coli FdrA Variant Derived from Syntrophic Coculture with a Methanogen Increases Succinate Production Due to Changes in Allantoin Degradation
Source: mSphere. 2021 Sep 8;6(5):e00654-21. doi: 10.1128/mSphere.00654-21 (PMC8550087; doi:10.1128/mSphere.00654-21)
Supplement: TABLE S1 [file msphere.00654-21-st001.docx]

Table S1.

|  |  | Ancestral *E. coli* | LMB061 (ΔlacZ) |
| --- | --- | --- | --- |
| Consumption  (mM) | Glycerol | 18.9±4.4*^a^* | 17.5±1.7 |
| Production  (mM) | Succinate | 2.5±0.3 | 2.2±0.1 |
|  | Formate | 0 | 0 |
|  | Acetate | 7.2±0.5 | 7.3±0.5 |
|  | Ethanol | 14.3±2.5 | 12.5±0.8 |
|  | Methane (ppm) | 6989±1472 | 7277±956 |
| Growth | OD_600_ | 1.06±0 | 1.04±0.1 |
| Succinate/cell mass (mM/gDW) | | 7.8±1.3 | 7.1±0.5 |

*^a^*, Values represent avg ± SD for 3 replicates. Unpaired two-tailed student’s t-tests were performed to analyze ancestral *E. coli* and LMB061. Statistical significance was defined as P < 0.05.
