# Supplementary material for: An Escherichia coli FdrA Variant Derived from Syntrophic Coculture with a Methanogen Increases Succinate Production Due to Changes in Allantoin Degradation
Source: mSphere. 2021 Sep 8;6(5):e00654-21. doi: 10.1128/mSphere.00654-21 (PMC8550087; doi:10.1128/mSphere.00654-21)
Supplement: TABLE S2 [file msphere.00654-21-st002.docx]

Table S2.

| Growth condition for *E. coli* | Ancestor | 5^th^ | 15^th^ | 20^th^ | 25^th^ | 30^th^ | 39^th^ | 43^rd^ |
| --- | --- | --- | --- | --- | --- | --- | --- | --- |
| Co-culture with *M. formicicum* on glycerol  under anaerobic condition | 1.05±0.11^A^ *^a b^* | 1.76±0.17^A^ | 2.13±0.08^A^ | 1.70±0.12^A^ | 1.53±0.20^A^ | 1.54±0.11^A^ | 1.61±0.04^A^ | 1.68±0.05^A^ |
| Single culture on glycerol + DMSO  Under anaerobic condition | 1.01±0.01^A^ | 1.18±0.04^B^ | 1.27±0.03^B^ | 1.45±0.13^B^ | 1.14±0.05^B^ | 0.97±0.04^B^ | 1.11±0.18^B^ | 1.29±0.19^B^ |
| Single culture in LB  Under aerobic condition | 1.01±0.01^A^ | 0.99±0.07^B^ | 0.96±0.03^C^ | 0.86±0.06^C^ | 0.88±0.02^C^ | 0.82±0.07^B^ | 0.91±0.08^B^ | 0.91±0.07^C^ |

*^a^*, The different superscripts in respective *E. coli* population indicate significant differences at p<0.05. SPSS PAWS Statistics, One-Way Anova, Duncan’s multiple comparisons.

*^b^*, Values represent avg ± SD for 3 replicates.
