# Supplementary material for: An Escherichia coli FdrA Variant Derived from Syntrophic Coculture with a Methanogen Increases Succinate Production Due to Changes in Allantoin Degradation
Source: mSphere. 2021 Sep 8;6(5):e00654-21. doi: 10.1128/mSphere.00654-21 (PMC8550087; doi:10.1128/mSphere.00654-21)
Supplement: TABLE S3 [file msphere.00654-21-st003.docx]

Table S3.

| Sample | Raw (base) | Unaligned (base) | Target aligned (base) | Depth of coverage |
| --- | --- | --- | --- | --- |
| Ancestor | 5,162,600,860 | 263,864,559 | 4,898,736,301 | 1059.11 |
| 5^th^ EM20M | 5,202,923,696 | 791,180,523 | 4,411,743,173 | 953.82 |
| 15^th^ EM20M | 4,955,970,414 | 1,024,519,800 | 3,931,450,614 | 849.98 |
| 20^th^ EM20M | 5,674,478,556 | 922,922,405 | 4,751,556,151 | 1027.29 |
| 25^th^ EM20M | 5,772,736,810 | 794,834,204 | 4,977,902,606 | 1076.22 |
| 30^th^ EM20M | 3,791,961,978 | 617,043,370 | 3,174,918,608 | 686.42 |
| 35^th^ EM20M | 3,265,373,026 | 546,100,851 | 2,719,272,175 | 587.91 |
| 39^th^ EM20M | 3,057,780,050 | 610,361,045 | 2,447,419,005 | 529.13 |
| 43^rd^ EM20M | 3,564,347,166 | 530,893,912 | 3,033,453,254 | 655.83 |
