# Supplementary material for: An Escherichia coli FdrA Variant Derived from Syntrophic Coculture with a Methanogen Increases Succinate Production Due to Changes in Allantoin Degradation
Source: mSphere. 2021 Sep 8;6(5):e00654-21. doi: 10.1128/mSphere.00654-21 (PMC8550087; doi:10.1128/mSphere.00654-21)
Supplement: TABLE S4 [file msphere.00654-21-st004.docx]

Table S4.

| Passages | 5^th^ | 15^th^ | 20^th^ | 25^th^ | 30^th^ | 35^th^ | 39^th^ | 43^rd^ |
| --- | --- | --- | --- | --- | --- | --- | --- | --- |
| Synonymous | 0 | 0 | 48 (0.625) | 47 (0.740) | 100 (0.419) | 99 (0.420) | 147 (0.311) | 72 (0.591) |
| Missense | 3 (0.461) | 7 (0.317) | 129 (0.573) | 123 (0.696) | 221 (0.448) | 220 (0.449) | 367 (0.312) | 158 (0.624) |
| Frameshift | 0 | 2 (0.142) | 9 (0.836) | 9 (0.943) | 21 (0.494) | 19 (0.527) | 33 (0.335) | 15 (0.598) |
| Nonsense | 0 | 0 | 9 (0.603) | 10 (0.609) | 15 (0.491) | 14 (0.521) | 14 (0.432) | 9 (0.642) |
