# Supplementary material for: An Escherichia coli FdrA Variant Derived from Syntrophic Coculture with a Methanogen Increases Succinate Production Due to Changes in Allantoin Degradation
Source: mSphere. 2021 Sep 8;6(5):e00654-21. doi: 10.1128/mSphere.00654-21 (PMC8550087; doi:10.1128/mSphere.00654-21)
Supplement: TABLE S5 [file msphere.00654-21-st005.docx]

Table S5.

| Mutation in gene  (b No.) | Glycerol (mM) | | | Fermentation products (mM) | | | | OD_600_ | pH | succinate yield (mM/gDW) | Protein (change in amino acid sequence) |
| --- | --- | --- | --- | --- | --- | --- | --- | --- | --- | --- | --- |
|  | added | | consumed | Succ*^a^* | For*^b^* | Ace*^c^* | EtOH*^d^* |  |  |  |  |
| *talB*  (b0008) | 82.3 | | 74.9 | 13.1 | 0 | 24.1 | 45.0 | 1.25 | 6.0 | 34.9 | transaldolase B (T33S) |
| *caiF*  (b0034) | 79.1 | | 40.2 | 5.1 | 0 | 7.7 | 34.4 | 1.26 | 6.6 | 13.5 | cai operon transcriptional activator (E67G) |
| *sgrR*  (b0069) | 84.6 | | 74.2 | 13.0 | 0 | 17.7 | 52.1 | 1.31 | 6.2 | 33.1 | transcriptional DNA-binding transcriptional activator of sgrS sRNA (H479Q) |
| *ycfS*  (b0113) | 93.3 | | 73.7 | 12.1 | 0 | 25.8 | 42.9 | 1.23 | 6.1 | 32.8 | (=*ldtC*), L,D-transpeptidase linking Lpp to murein (T227I) |
| *dnaQ*  (b0215) | 94.3 | | 9.0 | 2.1 | 0 | 4.7 | 10.6 | 1.30 | 7.1 | 1.6 | DNA polymerase III ε subunit (E14Q) |
| *ecpC*  (b0291) | 86.6 | | 69.8 | 11.8 | 0 | 18.5 | 48.4 | 1.30 | 6.2 | 30.1 | ECP production outer membrane protein (A27T) |
| *insEF-1*  (b0299) | 79.7 | | 35.2 | 4.4 | 0 | 7.5 | 29.4 | 0.85 | 6.8 | 17.3 | putative trasposase (C274Y) |
| *yahD*  (b0318) | 89.3 | | 76.2 | 12.8 | 0 | 25.3 | 45.8 | 1.26 | 6.0 | 33.9 | ankyrin repeat protein (H70Y) |
| *yahN^e^*  (b0328) | 86.2 | | 74.0 | 6.5 | 0 | 11.4 | 69.0 | 1.28 | 6.6 | 16.9 | amino acid exporter for proline, lysine, glutamate, homoserine (F136fs*^f^*) |
| *acpH*  (b0404) | 81.6 | | 28.0 | 4.2 | 0 | 6.3 | 23.8 | 1.28 | 6.9 | 10.9 | acyl carrier protein (ACP) phosphodiesterase; ACP hydrolyase (V109M) |
| *mdlA*  (b0448) | 87.8 | | 74.3 | 12.8 | 0 | 21.9 | 46.4 | 1.23 | 6.2 | 34.7 | putative multidrug ABC transporter ATPase (I311T) |
| *ybbN*  (b0492) | 80.8 | | 74.3 | 13.3 | 0 | 24.9 | 44.5 | 1.24 | 6.0 | 35.8 | DnaK co-chaperone, thioredoxin-like protein (P135L) |
| *cusC^e^* (b0572) | 86.8 | | 35.6 | 3.5 | 0 | 10.3 | 27.8 | 1.16 | 6.9 | 10.1 | copper/silver efflux system, outer membrane component (P306fs*^f^*) |
| *djlB*  (b0646) | 91.7 | | 75.2 | 12.6 | 0 | 21.0 | 48.4 | 1.24 | 6.3 | 33.9 | putative HscC co-chaperone, uncharacterized J domain-containing protein (E107K) |
| *ybgK*  (b0712) | 87.5 | | 70.5 | 11.9 | 0 | 20.7 | 45.0 | 1.26 | 6.3 | 31.5 | putative allophanate hydrolase, subunit 2 (R104L) |
| *cydA*  (b0733) | 79.4 | | 35.4 | 5.0 | 0 | 8.3 | 28.2 | 1.26 | 6.9 | 13.2 | cytochrome d terminal oxidase, subunit I (G56V) |
| *ybiO*  (b0808) | 86.2 | | 27.6 | 3.7 | 0 | 7.1 | 23.1 | 1.30 | 7.0 | 9.5 | mechanosensitive channel protein, intermediate conductance (F235S) |
| *ybjT*  (b0869) | 82.8 | | 23.5 | 3.1 | 0 | 6.3 | 20.2 | 0.68 | 6.9 | 15.2 | putative NAD-dependent oxidoreductase (E352K) |
| *ycaQ*  (b0916) | 78.1 | | 66.2 | 11.2 | 0 | 24.0 | 38.5 | 1.18 | 6.1 | 31.6 | winged helix DNA-binding domain-containing protein (R338L) |
| *ycaQ^e^*  (b0916) | 77.5 | | 37.0 | 2.7 | 0 | 8.7 | 32.3 | 0.91 | 6.8 | 9.9 | winged helix DNA-binding domain-containing protein (A240fs*^f^*) |
| *ycbF*  (b0944) | 90.6 | | 78.0 | 13.2 | 0 | 19.2 | 53.1 | 1.29 | 6.2 | 34.1 | putative periplasmic pilin chaperone (E62V) |
| *rutG*  (b1006) | 80.4 | | 65.8 | 11.7 | 0 | 23.5 | 37.6 | 1.15 | 6.1 | 33.9 | pyrimidine permease (A261V) |
| *rluC^e^*  (b1086) | 79.8 | | 31.0 | 2.4 | 0 | 10.6 | 23.3 | 0.66 | 6.8 | 12.1 | 23S rRNA pseudouridine(955,2504,2580) synthase (L195**^g^*) |
| *tfaE*  (b1156) | 91.2 | | 78.4 | 12.4 | 0 | 14.0 | 61.7 | 1.16 | 6.3 | 35.6 | e14 prophage; putative tail fiber assembly protein (A130D) |
| *dhaK*  (b1200) | 88.9 | | 81.1 | 8.7 | 0 | 17.5 | 63.6 | 1.36 | 6.5 | 21.4 | dihydroxyacetone kinase, PTS-dependent, dihydroxyacetone-binding subunit (A199E) |
| *fimZ*  (b1221) | 88.9 | | 83.0 | 12.6 | 0 | 12.8 | 67.3 | 1.26 | 6.4 | 33.2 | (=sfmZ),fimbrial Z protein; probable signal transducer (H193Y) |
| *rssA*  (b1234) | 81.2 | | 74.5 | 13.3 | 0 | 23.9 | 45.0 | 1.29 | 6.0 | 34.4 | putative patatin-like family phospholipase (G43C) |
| *adhE*  (b1241) | 85.4 | | 24.3 | 3.3 | 0 | 9.2 | 17.1 | 0.89 | 6.9 | 12.4 | iron-dependent alcohol dehydrogenase/pyruvate-formate lyase deactivase (P339Q) |
| *oppF^e^*  (b1247) | 76.9 | | 32.3 | 2.4 | 0 | 9.9 | 26.1 | 0.83 | 6.8 | 9.6 | oligopeptide ABC transporter ATPase (E139**^g^*) |
| *yciM*  (b1280) | 88.7 | | 14.8 | 10.7 | 0 | 9.1 | 0 | 0.37 | 6.5 | 96.4 | LPS regulatory protein; putative modulator of LpxC proteolysis (K169N) |
| *osmB*  (b1283) | 81.9 | | 20.1 | 2.6 | 0 | 5.6 | 17.8 | 1.14 | 6.9 | 7.6 | osmotically and stress inducible lipoprotein (M19R) |
| *gmr*  (b1285) | 88.7 | | 80.6 | 13.2 | 0 | 26.5 | 47.2 | 1.26 | 5.9 | 34.9 | cyclic-di-GMP phosphodiesterase; csgD regulator; modulator of RNase II stability (H97Y) |
| *sapA*  (b1294) | 90.2 | | 78.0 | 12.9 | 0 | 13.6 | 61.3 | 1.18 | 6.4 | 36.4 | antimicrobial peptide transport ABC transporter periplasmic binding protein (F65L) |
| *ydbK*  (b1378) | 82.3 | | 73.5 | 13.0 | 0 | 24.4 | 43.1 | 1.22 | 6.0 | 35.5 | (=*pfo*), pyruvate-flavodoxin oxidoreductase (V598E) |
| *yncD*  (b1451) | 91.2 | | 74.4 | 13.1 | 0 | 13.6 | 57.8 | 1.27 | 6.4 | 34.4 | putative iron outer membrane transporter (G504S) |
| *ydcC^e^*  (b1460) | 83.3 | | 30.8 | 2.6 | 0 | 9.1 | 25.2 | 1.05 | 6.8 | 8.3 | H repeat-associated putative transposase (Q187**^g^*) |
| *ddpX^e^*  (b1488) | 88.3 | | 21.8 | 2.3 | 0 | 7.3 | 17.7 | 1.05 | 6.9 | 7.3 | D-ala-D-ala dipeptidase, Zn-dependent (T5fs*^f^*) |
| *gadC*  (b1492) | 86.3 | | 35.8 | 4.7 | 0 | 8.1 | 29.0 | 0.90 | 6.8 | 17.4 | glutamate:gamma-aminobutyric acid antiporter (A257E) |
| *sufD^e^*  (b1681) | 86.4 | | 43.4 | 3.6 | 0 | 9.5 | 36.9 | 0.97 | 6.8 | 12.4 | component of SufBCD Fe-S cluster assembly scaffold (G338fs*^f^*) |
| *yoaB*  (b1809) | 82.2 | | 67.5 | 10.4 | 0 | 24.2 | 40.2 | 1.25 | 6.2 | 27.7 | putative reactive intermediate deaminase (A68S) |
| *manX*  (b1817) | 78.3 | | 36.3 | 3.7 | 0 | 7.1 | 32.2 | 1.06 | 6.8 | 11.6 | fused mannose-specific PTS enzymes: IIA component/IIB component (L207R) |
| *motA*  (b1890) | 79.2 | | 23.5 | 2.9 | 0 | 6.4 | 20.1 | 0.99 | 6.9 | 9.8 | proton conductor component of flagella motor (E157K) |
| *yeeO*  (b1985) | 81.0 | | 70.2 | 11.9 | 0 | 24.6 | 40.6 | 1.21 | 6.2 | 32.8 | putative multdrug exporter, MATE family (I331N) |
| *ugd*  (b2028) | 82.4 | | 72.5 | 12.9 | 0 | 22.5 | 44.5 | 1.22 | 6.2 | 35.2 | UDP-glucose 6-dehydrogenase (K295Q) |
| *yegR^e^*  (b2085) | 83.4 | | 47.2 | 4.8 | 0 | 9.8 | 39.7 | 0.99 | 6.8 | 16.2 | uncharacterized protein (E3fs*^f^*) |
| *yejO*  (b2192) | 85.6 | | 64.3 | 10.4 | 0 | 12.0 | 49.0 | 1.35 | 6.42 | 25.7 | pseudogene, autotransporter outer membrane ;putative transport (D511Y) |
| *nuoM*  (b2277) | 80.3 | | 28.2 | 3.2 | 0 | 6.9 | 24.1 | 1.01 | 6.9 | 10.6 | NADH:ubiquinone oxidoreductase, membrane subunit M (A32S) |
| *ypjM*  (b2641) | 92.2 | | 24.9 | 3.3 | 0 | 12.6 | 14.5 | 1.24 | 6.6 | 8.9 | CP4-57 prophage; putative arsenite transporter, N-terminal fragment (G153R) |
| *yqaE*  (b2666) | 92.5 | | 30.2 | 3.7 | 0 | 13.5 | 19.1 | 0.93 | 6.8 | 13.3 | cyaR sRNA-regulated protein (R51L) |
| *hypD*  (b2729) | 80.6 | | 30.5 | 4.8 | 0 | 7.0 | 24.4 | 1.08 | 6.9 | 14.9 | hydrogenase maturation protein (H26Q) |
| *speC*  (b2965) | 92.5 | | 69.5 | 10.7 | 0 | 13.2 | 52.9 | 1.40 | 6.38 | 25.5 | ornithine decarboxylase, constitutive (R258H) |
| *yqgA*  (b2966) | 93.7 | | 28.1 | 3.1 | 0 | 12.0 | 16.1 | 0.94 | 6.9 | 11.0 | DUF554 family putative inner membrane protein (S33F) |
| *ygiS*  (b3020) | 97.4 | | 71.3 | 11.1 | 0 | 12.7 | 55.8 | 1.40 | 6.41 | 26.4 | putative ABC transporter permease (G44S) |
| *yqjA*  (b3095) | 91.1 | | 17.2 | 2.0 | 0 | 6.0 | 14.8 | 1.11 | 7.0 | 6.0 | general envelope maintenance protein; putative multidrug efflux transporter (S160R) |
| *yrbL*  (b3207) | 78.4 | | 28.9 | 3.4 | 0 | 13.7 | 17.7 | 0.98 | 6.5 | 11.6 | Mg^2+^-starvation-stimulated protein (R198C) |
| *yhdW*  (b3268) | 83.4 | | 61.7 | 9.7 | 0 | 22.4 | 36.8 | 1.23 | 6.4 | 26.3 | predicted amino-acid transporter subunit (G285C) |
| *rsmB^e^*  (b3289) | 80.9 | | 50.5 | 3.6 | 0 | 9.1 | 44.6 | 1.27 | 6.7 | 9.4 | 16S rRNA m(5)C967 methyltransferase, SAM-dependent (Q309**^g^*) |
| *rpsD*  (b3296) | 86.5 | | 73.6 | 11.7 | 0 | 11.7 | 59.5 | 1.23 | 6.4 | 31.7 | 30S ribosomal subunit protein S4 (R10H) |
| *fusA*  (b3340) | 84.1 | | 70.7 | 12.0 | 0 | 10.3 | 59.6 | 1.17 | 6.3 | 34.0 | protein chain elongation factor EF-G, GTP-binding (A552P) |
| *envZ^e^*  (b3404) | 90.5 | | 17.8 | 2.6 | 0 | 6.9 | 13.6 | 1.16 | 6.9 | 7.5 | sensory histidine kinase in two-component regulatory system with OmpR (I350fs*^f^*) |
| *rlmJ*  (b3499) | 86.5 | | 17.3 | 12.2 | 0 | 9.7 | 0 | 0.52 | 6.7 | 77.9 | 23S rRNA m(6)A2030 methyltransferase, SAM-dependent (E77G) |
| *yhiD^e^*  (b3508) | 80.2 | | 37.6 | 2.8 | 0 | 9.5 | 30.7 | 1.01 | 6.8 | 9.2 | putative Mg^2+^ transport ATPase, inner membrane protein (M25fs*^f^*) |
| *kdgK^e^*  (b3526) | 81.7 | | 53.2 | 3.5 | 0 | 12.3 | 44.4 | 1.24 | 6.7 | 9.4 | 2-dehydro-3-deoxygluconokinase (N82fs*^f^*) |
| *setC*  (b3659) | 82.2 | | 70.4 | 11.7 | 0 | 24.3 | 40.4 | 1.18 | 6.1 | 33.1 | putative arabinose efflux transporter (R217W) |
| *ilvA^e^*  (b3772) | 81.8 | | 26.8 | 2.9 | 0 | 8.9 | 20.0 | 0.99 | 6.9 | 9.8 | l-threonine dehydratase, biosynthetic; also known as threonine deaminase (E332**^g^*) |
| *glpK*  (b3926) | | 87.1 | 6.2 | 0.7 | 0 | 4.3 | 6.3 | 0.92 | 7.1 | 2.5 | glycerol kinase (K173N) |
| *gldA*  (b3945) | | 81.5 | 38.5 | 9.5 | 0 | 14.2 | 20.2 | 1.09 | 6.5 | 29.1 | glycerol dehydrogenase, NAD^+^ dependent; 1,2-propanediol:NAD^+^ oxidoreductase (I113N) |
| *fsaB*  (b3946) | | 84.3 | 83.1 | 12.6 | 0 | 11.9 | 67.0 | 1.32 | 6.4 | 31.8 | fructose-6-phosphate aldolase 2 (A199V) |
| *thiC*  (b3994) | 87.3 | | 76.5 | 13.1 | 0 | 25.0 | 46.4 | 1.29 | 6.0 | 33.9 | phosphomethylpyrimidine synthase (E407K) |
| *blc*  (b4149) | 87.5 | | 74.4 | 9.8 | 0 | 10.1 | 67.0 | 1.35 | 6.6 | 24.2 | outer membrane lipoprotein cell division and growth lipocalin (F109C) |
| *mscM*  (b4159) | 87.6 | | 27.1 | 3.3 | 0 | 6.4 | 23.8 | 1.28 | 7.0 | 8.6 | mechanosensitive channel protein, miniconductance (R158C) |
| *treB*  (b4240) | 81 | | 73.6 | 13.1 | 0 | 23.8 | 44.5 | 1.18 | 6.1 | 37.0 | trehalose-specific PTS enzyme: IIB and IIC component (V113E) |
| *uxuB*  (b4323) | 85.1 | | 33.1 | 3.7 | 0 | 7.6 | 26.8 | 1.31 | 6.9 | 9.4 | D-mannonate oxidoreductase, NAD-dependent (G131C) |
| *yjiT*  (b4342) | 93.4 | | 81.1 | 12.6 | 0 | 27.9 | 48.7 | 1.28 | 5.9 | 32.8 | Pseudogene (D389E) |
| *yjjA^e^*  (b4360) | 84.8 | | 76.4 | 6.0 | 0 | 10.8 | 69.3 | 1.29 | 6.6 | 15.5 | putative DUF2501 family periplasmic protein (Q86**^g^*) |
| *gntU*  (b4476) | 88.1 | | 82.4 | 11.3 | 0 | 12.6 | 68.1 | 1.24 | 6.4 | 30.5 | gluconate transporter, low affinity GNT 1 system (A101S) |
| *ydbJ*  (b4529) | 88.4 | | 72.3 | 11.4 | 0 | 27.4 | 41.1 | 1.20 | 5.8 | 31.7 | DUF333 family putative lipoprotein (S37L) |

*^a^*, Succinate.

*^b^*, Formate.

*^c^*, Acetate.

*^d^*, Ethanol.

*^e^*, Mutant *E. coli* with nonsense variants. The remains are mutant *E. coli* with a SNP.

*^f^*, Frame shift.

*^g^*, Stop codon.
