# Supplementary material for: An Escherichia coli FdrA Variant Derived from Syntrophic Coculture with a Methanogen Increases Succinate Production Due to Changes in Allantoin Degradation
Source: mSphere. 2021 Sep 8;6(5):e00654-21. doi: 10.1128/mSphere.00654-21 (PMC8550087; doi:10.1128/mSphere.00654-21)
Supplement: TABLE S6 [file msphere.00654-21-st006.docx]

Table S6.

| Growth condition for *E. coli* | *cysN* | *dnaK* | *fdrA* | *pgpC* | *rob* | *ybbP* | *yfjI* |
| --- | --- | --- | --- | --- | --- | --- | --- |
| Co-culture with *M. formicicum* on glycerol  under anaerobic condition | 1.03±0.16^A^ *^a b^* | 0.82±0.21^A^ | 0.85±0.18^AB^ | 1.18±0.22^A^ | 0.93±0.06^AB^ | 0.90±0.13^A^ | 0.99±0.18^A^ |
| Single culture on glycerol + DMSO  Under anaerobic condition | 1.00±0.12^A^ | 1.10±0.24^A^ | 0.56±0.10^A^ | 0.84±0.12^B^ | 0.78±0.21^A^ | 0.96±0.03^AB^ | 0.88±0.23^A^ |
| Single culture in LB  Under aerobic condition | 1.18±0.14^A^ | 1.11±0.17^A^ | 1.12±0.18^B^ | 1.02±0.07^AB^ | 1.21±0.12^B^ | 1.14±0.09^B^ | 1.09±0.05^A^ |

*^a,^* The different superscripts in respective *E. coli* population indicate significant differences at p<0.05. SPSS PAWS Statistics, One-Way Anova, Duncan’s multiple comparisons.

*^b^*, Values represent avg ± SD for 3 replicates.
