# Supplementary material for: An Escherichia coli FdrA Variant Derived from Syntrophic Coculture with a Methanogen Increases Succinate Production Due to Changes in Allantoin Degradation
Source: mSphere. 2021 Sep 8;6(5):e00654-21. doi: 10.1128/mSphere.00654-21 (PMC8550087; doi:10.1128/mSphere.00654-21)
Supplement: TABLE S7 [file msphere.00654-21-st007.docx]

Table S7.

A.

| Subtrate | *cysN* | *dnaK* | *fdrA* | *rob* | *ybbP* | *yfjI* | *pgpC* |
| --- | --- | --- | --- | --- | --- | --- | --- |
| Glycerol | 1.023 | 1.038 | 1.027 | 1.033 | 1.040 | 1.024 | 1.003 |
| Xylose | 0.997 | 1.007 | 0.999 | 0.991 | 1.013 | 0.995 | 0.996 |
| Glucose | 1.002 | 1.006 | 0.970 | 0.994 | 0.983 | 0.995 | 0.998 |
| Succinate | 1.019 | 1.029 | 0.986 | 0.998 | 1.010 | 1.016 | 1.044 |

B.

| Subtrate | *cysN* | *dnaK* | *fdrA* | *rob* | *ybbP* | *yfjI* | *pgpC* |
| --- | --- | --- | --- | --- | --- | --- | --- |
| Glycerol + DMSO | 0.999 | 1.040 | 1.014 | 1.028 | 0.997 | 1.037 | 0.988 |
| Xylose | 1.002 | 1.004 | 0.997 | 0.999 | 1.000 | 0.988 | 1.000 |
| Glucose | 0.996 | 0.999 | 1.004 | 1.017 | 1.007 | 0.995 | 0.999 |
| Glycerol + fumarate | 0.989 | 1.002 | 1.002 | 0.995 | 1.003 | 0.989 | 0.993 |

C.

a.

| Time (h) | Ancestor | 39^th^ | *cysN* | *dnaK* | *fdrA* | *rob* | *ybbP* | *yfjI* | *pgpC* |
| --- | --- | --- | --- | --- | --- | --- | --- | --- | --- |
| 0 | 0.013 | 0.018 | 0.005 | 0.010 | 0.013 | 0.011 | 0.016 | 0.012 | 0.007 |
| 1 | 0.027 | 0.044 | 0.018 | 0.026 | 0.041 | 0.030 | 0.046 | 0.029 | 0.025 |
| 2 | 0.074 | 0.075 | 0.058 | 0.068 | 0.085 | 0.064 | 0.087 | 0.073 | 0.067 |
| 3 | 0.119 | 0.105 | 0.100 | 0.116 | 0.134 | 0.114 | 0.133 | 0.131 | 0.116 |
| 4 | 0.163 | 0.140 | 0.133 | 0.164 | 0.184 | 0.152 | 0.177 | 0.173 | 0.145 |
| 5 | 0.200 | 0.184 | 0.177 | 0.224 | 0.237 | 0.204 | 0.232 | 0.222 | 0.173 |
| 6 | 0.254 | 0.244 | 0.218 | 0.275 | 0.308 | 0.247 | 0.286 | 0.272 | 0.207 |
| 7 | 0.292 | 0.325 | 0.253 | 0.328 | 0.353 | 0.280 | 0.326 | 0.306 | 0.247 |
| 8 | 0.329 | 0.338 | 0.289 | 0.363 | 0.406 | 0.325 | 0.366 | 0.337 | 0.275 |
| 24 | 0.789 | 0.725 | 0.514 | 0.648 | 0.612 | 0.531 | 0.665 | 0.671 | 0.562 |

b.

| Time (h) | Ancestor | 39^th^ | *cysN* | *dnaK* | *fdrA* | *rob* | *ybbP* | *yfjI* | *pgpC* |
| --- | --- | --- | --- | --- | --- | --- | --- | --- | --- |
| 0 | 0.007 | 0.022 | 0.005 | 0.008 | 0.010 | 0.106 | 0.016 | 0.015 | 0.011 |
| 1 | 0.027 | 0.044 | 0.018 | 0.026 | 0.041 | 0.030 | 0.046 | 0.029 | 0.025 |
| 2 | 0.074 | 0.090 | 0.065 | 0.090 | 0.079 | 0.235 | 0.136 | 0.102 | 0.097 |
| 3 | 0.146 | 0.146 | 0.134 | 0.162 | 0.149 | 0.277 | 0.207 | 0.174 | 0.166 |
| 4 | 0.217 | 0.191 | 0.193 | 0.221 | 0.205 | 0.340 | 0.272 | 0.232 | 0.221 |
| 5 | 0.306 | 0.267 | 0.266 | 0.314 | 0.305 | 0.394 | 0.349 | 0.328 | 0.290 |
| 6 | 0.399 | 0.353 | 0.332 | 0.387 | 0.386 | 0.448 | 0.431 | 0.403 | 0.370 |
| 7 | 0.501 | 0.442 | 0.382 | 0.446 | 0.456 | 0.525 | 0.525 | 0.462 | 0.469 |
| 8 | 0.592 | 0.524 | 0.435 | 0.498 | 0.513 | 0.592 | 0.583 | 0.508 | 0.528 |
| 24 | 0.793 | 0.780 | 0.652 | 0.641 | 0.773 | 0.904 | 0.617 | 0.732 | 0.528 |

c.

| Time (h) | Ancestor | 39^th^ | *cysN* | *dnaK* | *fdrA* | *rob* | *ybbP* | *yfjI* | *pgpC* |
| --- | --- | --- | --- | --- | --- | --- | --- | --- | --- |
| 0 | 0.008 | 0.004 | 0.002 | 0.011 | 0.006 | 0.026 | 0.018 | 0.009 | 0.001 |
| 1 | 0.039 | 0.029 | 0.041 | 0.076 | 0.039 | 0.059 | 0.060 | 0.060 | 0.037 |
| 2 | 0.105 | 0.084 | 0.116 | 0.157 | 0.092 | 0.144 | 0.129 | 0.146 | 0.102 |
| 3 | 0.242 | 0.195 | 0.246 | 0.292 | 0.193 | 0.334 | 0.257 | 0.335 | 0.270 |
| 4 | 0.469 | 0.349 | 0.472 | 0.538 | 0.363 | 0.573 | 0.490 | 0.574 | 0.498 |
| 5 | 0.753 | 0.575 | 0.741 | 0.788 | 0.661 | 0.845 | 0.758 | 0.843 | 0.787 |
| 6 | 0.902 | 0.727 | 0.881 | 0.922 | 0.843 | 0.997 | 0.912 | 1.010 | 1.003 |
| 7 | 1.040 | 0.880 | 0.996 | 0.997 | 0.980 | 1.130 | 1.003 | 1.113 | 1.039 |
| 8 | 1.108 | 1.003 | 1.033 | 1.010 | 1.009 | 1.160 | 1.031 | 1.118 | 1.063 |
| 24 | 0.857 | 0.848 | 0.958 | 0.795 | 0.866 | 1.034 | 0.842 | 0.894 | 0.987 |

d.

| Time (h) | Ancestor | 39^th^ | *cysN* | *dnaK* | *fdrA* | *rob* | *ybbP* | *yfjI* | *pgpC* |
| --- | --- | --- | --- | --- | --- | --- | --- | --- | --- |
| 0 | 0.009 | 0.014 | 0.046 | 0.012 | 0.016 | 0.112 | 0.054 | 0.012 | 0.004 |
| 1 | 0.039 | 0.029 | 0.041 | 0.076 | 0.039 | 0.059 | 0.060 | 0.060 | 0.033 |
| 2 | 0.086 | 0.080 | 0.138 | 0.096 | 0.092 | 0.224 | 0.129 | 0.080 | 0.070 |
| 3 | 0.122 | 0.110 | 0.159 | 0.130 | 0.121 | 0.260 | 0.160 | 0.118 | 0.106 |
| 4 | 0.150 | 0.138 | 0.181 | 0.157 | 0.149 | 0.299 | 0.185 | 0.142 | 0.127 |
| 5 | 0.177 | 0.175 | 0.205 | 0.184 | 0.183 | 0.353 | 0.226 | 0.160 | 0.141 |
| 6 | 0.196 | 0.202 | 0.223 | 0.202 | 0.204 | 0.381 | 0.254 | 0.186 | 0.162 |
| 7 | 0.220 | 0.250 | 0.249 | 0.233 | 0.240 | 0.424 | 0.288 | 0.213 | 0.187 |
| 8 | 0.243 | 0.286 | 0.272 | 0.258 | 0.266 | 0.456 | 0.319 | 0.234 | 0.203 |
| 24 | 0.538 | 0.644 | 0.463 | 0.488 | 0.506 | 0.653 | 0.499 | 0.523 | 0.409 |

D.

a.

| Time (h) | Ancestor | 39^th^ | *cysN* | *dnaK* | *fdrA* | *rob* | *ybbP* | *yfjI* | *pgpC* |
| --- | --- | --- | --- | --- | --- | --- | --- | --- | --- |
| 0 | 0.007 | 0.005 | 0.001 | 0.001 | 0.008 | 0.008 | 0.016 | 0.015 | 0.021 |
| 8 | 0.275 | 0.193 | 0.247 | 0.202 | 0.231 | 0.236 | 0.230 | 0.278 | 0.233 |
| 24 | 0.879 | 0.904 | 0.767 | 0.723 | 0.892 | 0.684 | 0.726 | 0.736 | 0.718 |

b.

| Time (h) | Ancestor | 39^th^ | *cysN* | *dnaK* | *fdrA* | *rob* | *ybbP* | *yfjI* | *pgpC* |
| --- | --- | --- | --- | --- | --- | --- | --- | --- | --- |
| 0 | 0.036 | 0.027 | 0.100 | 0.016 | 0.009 | 0.008 | 0.013 | 0.001 | 0.049 |
| 8 | 0.491 | 0.480 | 0.554 | 0.433 | 0.452 | 0.406 | 0.415 | 0.401 | 0.466 |
| 24 | 0.797 | 0.848 | 0.734 | 0.578 | 0.716 | 0.604 | 0.604 | 0.634 | 0.592 |

c.

| Time (h) | Ancestor | 39^th^ | *cysN* | *dnaK* | *fdrA* | *rob* | *ybbP* | *yfjI* | *pgpC* |
| --- | --- | --- | --- | --- | --- | --- | --- | --- | --- |
| 0 | 0.077 | 0.056 | 0.162 | 0.018 | 0.007 | 0.013 | 0.014 | 0.044 | 0.018 |
| 8 | 1.066 | 0.921 | 1.254 | 0.996 | 0.933 | 0.959 | 0.913 | 0.967 | 0.872 |
| 24 | 0.795 | 0.930 | 1.152 | 0.807 | 0.711 | 0.680 | 0.771 | 0.790 | 0.679 |

d.

| Time (h) | Ancestor | 39^th^ | *cysN* | *dnaK* | *fdrA* | *rob* | *ybbP* | *yfjI* | *pgpC* |
| --- | --- | --- | --- | --- | --- | --- | --- | --- | --- |
| 0 | 0.015 | 0.011 | 0.049 | 0.004 | 0.003 | 0.003 | 0.008 | 0.031 | 0.009 |
| 8 | 0.371 | 0.411 | 0.415 | 0.340 | 0.255 | 0.340 | 0.312 | 0.356 | 0.347 |
| 24 | 1.109 | 1.127 | 0.801 | 0.680 | 0.676 | 0.787 | 0.663 | 0.642 | 0.803 |
